# Metabolomic profiling of upper GI malignancies in blood and tissue: a systematic review and meta-analysis

**DOI:** 10.1007/s00432-024-05857-5

**Published:** 2024-07-01

**Authors:** Ilja Balonov, Minca Mattis, Stefanie Jarmusch, Berthold Koletzko, Kathrin Heinrich, Jens Neumann, Jens Werner, Martin K. Angele, Christian Heiliger, Sven Jacob

**Affiliations:** 1 Department of General, Visceral and Transplantation Surgery, University Hospital, LMU Munich, Ludwig-Maximilians-University (LMU) Munich, Marchioninistr. 15, 81377 Munich, Germany; 2 Division of Metabolic and Nutritional Medicine, Dr. Von Hauner Children’s Hospital, Ludwig-Maximilians-University Munich Medical Center, Lindwurmstraße 4, 80337 Munich, Germany; 3 Department of Medicine III, University Hospital, LMU Munich, Munich, Germany; 4 Institute of Pathology, Ludwig-Maximilians-University (LMU) Munich, Munich, Germany

**Keywords:** Gastric cancer, Esophageal cancer, Cancer of the gastroesophageal junction, Tumour microenvironment, Metabolomics, Metabolomic profile, Oncometabolomic, Mass spectrometry, Meta-analysis

## Abstract

**Objective:**

To conduct a systematic review and meta-analysis of case–control and cohort human studies evaluating metabolite markers identified using high-throughput metabolomics techniques on esophageal cancer (EC), cancer of the gastroesophageal junction (GEJ), and gastric cancer (GC) in blood and tissue.

**Background:**

Upper gastrointestinal cancers (UGC), predominantly EC, GEJ, and GC, are malignant tumour types with high morbidity and mortality rates. Numerous studies have focused on metabolomic profiling of UGC in recent years. In this systematic review and meta-analysis, we have provided a collective summary of previous findings on metabolites and metabolomic profiling associated with EC, GEJ and GC.

**Methods:**

Following the PRISMA procedure, a systematic search of four databases (Embase, PubMed, MEDLINE, and Web of Science) for molecular epidemiologic studies on the metabolomic profiles of EC, GEJ and GC was conducted and registered at PROSPERO (CRD42023486631). The Newcastle–Ottawa Scale (NOS) was used to benchmark the risk of bias for case-controlled and cohort studies. QUADOMICS, an adaptation of the QUADAS-2 (Quality Assessment of Diagnostic Accuracy) tool, was used to rate diagnostic accuracy studies. Original articles comparing metabolite patterns between patients with and without UGC were included. Two investigators independently completed title and abstract screening, data extraction, and quality evaluation. Meta-analysis was conducted whenever possible. We used a random effects model to investigate the association between metabolite levels and UGC.

**Results:**

A total of 66 original studies involving 7267 patients that met the required criteria were included for review. 169 metabolites were differentially distributed in patients with UGC compared to healthy patients among 44 GC, 9 GEJ, and 25 EC studies including metabolites involved in glycolysis, anaerobic respiration, tricarboxylic acid cycle, and lipid metabolism. Phosphatidylcholines, eicosanoids, and adenosine triphosphate were among the most frequently reported lipids and metabolites of cellular respiration, while BCAA, lysine, and asparagine were among the most commonly reported amino acids. Previously identified lipid metabolites included saturated and unsaturated free fatty acids and ketones. However, the key findings across studies have been inconsistent, possibly due to limited sample sizes and the majority being hospital-based case–control analyses lacking an independent replication group.

**Conclusion:**

Thus far, metabolomic studies have provided new opportunities for screening, etiological factors, and biomarkers for UGC, supporting the potential of applying metabolomic profiling in early cancer diagnosis. According to the results of our meta-analysis especially BCAA and TMAO as well as certain phosphatidylcholines should be implicated into the diagnostic procedure of patients with UGC. We envision that metabolomics will significantly enhance our understanding of the carcinogenesis and progression process of UGC and may eventually facilitate precise oncological and patient-tailored management of UGC.

**Supplementary Information:**

The online version contains supplementary material available at 10.1007/s00432-024-05857-5.

## Introduction

Surgery of upper gastrointestinal cancers (UGC) such as esophageal cancer (EC), cancer of the gastroesophageal junction (GEJ) and gastric cancer (GC) are still one the most lethal types of cancer worldwide (Jin et al. 2020). Advances in surgical technique and perioperative systemic therapies have made many patients with more advanced disease or co-morbidities candidates for surgery (Bray et al. 2018; Perera et al. 2021; Siegel et al. 2022). In light of these changes, reliable measures that assess preoperative diagnostics, patient-centred outcomes and individualised follow-up tools are more important than ever to establish a precision oncology approach in diagnostics and multimodality therapy (Warburg et al. 1927; Slankamenac et al. 2013). Ideally, these measures will help guide treatment decisions and ensure treatment meets appropriate standards. Early detection and timely treatment following precise risk classification are imperative for improving patient outcomes in GC. The most frequent symptoms of UGC are very unspecific, like dysphagia, nausea, and loss of weight (Heslin, et al. 1997). The primary diagnostic tool is gastroscopy, an invasive procedure allowing tumour detection and biopsy collection in the same procedure (Heiliger et al. 2022). Conventional plasma tumour biomarkers have been proposed as valuable parameters for early detection, prognostic prediction and recurrence monitoring in EC and GC (Emoto et al. 2012). Nonetheless, because of limited specificity and sensitivity, most circulating molecular markers are not recommended for early diagnosis of UGC. Therefore, identification and validation of UGC-specific biomarkers are warranted to facilitate early diagnosis (Gurdasani et al. 2019). It is necessary to observe the progression from precancerous lesions to malignancies, and therefore, identifying diagnostic biomarkers for EC, GEJ, and GC is of great significance. A key feature of tumour cell metabolism is the ability to obtain nutrients from a frequently nutrient-poor microenvironment and utilise these nutrients to meet the demands of growth and proliferation (Lewis et al. 2021). Thereby a tumour microenvironment (TME) is sequentially formed, which comprises cancer cells, the cytokine environment, extracellular matrix, immune cell subsets and other components (Gustafsson et al. 2024). In this complex network of cancer cells and cancer derived metabolites, the pro-tumorigenic vicinity plays a pivotal role in stimulating tumour angiogenesis, promoting its invasiveness and metastatic potential (Berrell 2023). Certain tumour-associated metabolites are associated with aggressive cancer phenotypes, facilitated angiogenesis, promoted mutagenesis and suppress the immune system (Zhang et al. 2023). However, the migration of cancer derived metabolites from the tumour itself, as well as its microenvironment into the systemic blood circulation can be detected and quantified by state-of-the-art clinical mass spectrometry (Zhao et al. 2023).

For decades, metabolic reprogramming of tumours was perceived as only increased glycolysis, as postulated by Otto Warburg almost a century ago. This simplistic view has recently been challenged and revised as we realized that tumour metabolism is more heterogeneous than initially assumed (Thompson et al. 2023). The concept of metabolic plasticity has recently emerged as we learned that some tumours are able to switch between alternative metabolic programs to meet challenges exerted by drugs targeted against a particular metabolic pathway or during tumorigenesis (Finley 2023). It is suggested that EC and GC cells may consume a large number of fatty acids to meet the needs of cell membrane synthesis and energy production (Warburg et al. 1927). The survival of cancer cells in the human body depends on lipids, and accumulated lipid droplets are found in various cancer microenvironments (Mikami et al. 2019), so lipid droplets are expected as effective targets for blocking tumour growth (Petan et al. 2018), and fatty acid metabolism-related proteins may also become diagnostic markers for early GC diagnosis and follow-up (Wu et al. 2019; Jiang et al. 2017). In addition, UGC is prone to metastasis, and adipocytes regulate fatty acid oxidation. However, fatty acid oxidation is enhanced in UGC patients, which promotes metastasis in EC, GEJ, and GC (Tsuboi 2019; Tan et al. 2018). The plasma is commonly considered a pool of metabolites and reflects the systemic metabolic regulation in cancer patients (Lopez-Bascon et al. 2016). Multiple methods have been developed for metabolome assessment, including nuclear magnetic resonance spectroscopy (NMR), liquid chromatography (LC), gas chromatography, capillary electrophoresis (CE), and mass spectroscopy (MS). To date, liquid chromatography-tandem mass spectroscopy (LC–MS/MS)-based high throughput techniques are broadly utilised in various compartments, allowing joint assessment of multiple metabolites, only requiring small amounts of biological specimens (Chan et al. 2014).

Respectively, these “omics” technologies enable the study of cancer-related alterations at both a genetic level and at proteomic and metabolomic levels with high sensitivity (Doshi et al. 2023). Among these techniques, metabolomics is a promising approach for cancer biomarker discovery, as the reprogramming of cellular metabolism is one of the hallmarks of cancer, and metabolomics as the endpoint of “omics” cascades could reflect perturbations in all biological activities with an amplified way (Stine et al. 2022). Since the beginning of metabolomic research, guidelines have been defined by the Metabolomics Standards Initiative for data reporting, outlining the minimal information content that should be reported, common syntax, defining the transmission formats that facilitate the exchange of information, and standard semantics (Sumner et al. 2007). Thus, current metabolomic research provides global data on the metabolism of tumours and can also act as a promising tool to discover biomarkers of diagnosis, metastatic surveillance, and prediction of chemotherapeutic sensitivity (Warburg 1956a).

According to *The Human Metabolome Database* (HMDB), lipids, carbohydrates and amino acids constitute obligate cofactors in mitochondrial fatty acid -oxidation, which represents the major step of energy production and is altered in multiple malignancies (Lu et al. 2019; Wishart, et al. 2022). On the example of lipids, excessive production of acylcarnitines has been shown to reflect altered fatty acid oxidation, contributing to metabolic diseases (Balonov, et al. 2023). In cancer, acylcarnitine metabolism is considered a gridlock to precisely promote metabolic flexibility based on its crucial function in controlling the metabolic processes of glucose and fatty acids (Warburg 1956b). Metabolic reprogramming in malignant cells modulates the production of acylcarnitines of different chain lengths (Kim et al. 2023). This intercalates with other major metabolic pathways, factors and metabolites, resulting in balanced energy production and consumption, as well as in the biosynthesis of metabolic intermediates for fast growth (Liu et al. 2018). However, studies evaluating acylcarnitine profiles associated with EC, GEJ and GC are scarce and inconsistent (Sun et al. 2023). Corona et al. found that plasma acetylcarnitine (C2), hexadecanoylcarnitine (C16) and octadecenoylcarnitine (C18:1) of UGC patients were higher than first-degree relatives (Corona, et al. 2018). A study performed by Lario et al. found that plasma hydroxytetradecadienylcarnitine (C14:2-OH) and octadecanoylcarnitine (C18) were increased in GC patients compared to precursor lesions of gastric cancer (Lario et al. 2017). Therefore, more studies are required to assess metabolic changes in UGC.

## Methods

This systematic review was developed, conducted and reported following the Preferred Reporting Items for Systematic Reviews and Meta-Analyses (PRISMA) guidelines and relevant recommendations; Good Clinical Practice (GCP)-compliant handling of the data is secured by adequate SOPs (Page et al. 2021; Nagendrababu et al. 2020). The review protocol was established before the initiation of the research and registered in the International Prospective Register of Systematic Reviews (PROSPERO) (CRD42023486631) (Schiavo 2019).

### Literature search strategy

A systematic literature search was conducted using PubMed, Embase, Web of Science, and MEDLINE according to the PRISMA guidelines by two independent investigators (IB and SJ) (Shamseer et al. 2015). All studies published in English through January 2023 were potentially eligible for inclusion. We have performed searches using the keywords (metabolomics OR metabolomic profiling OR metabolomic fingerprinting OR oncomet) AND (gastric cancer OR cancer of the gastroesophageal junction OR esophageal cancer) present in the [Title] or [Title/Abstract], as well as combinations, and limits (humans, adults, English language etc.). The reference list of each article was searched for further relevant literature. Duplicate articles, editorials, and conference abstracts were excluded. The articles were screened and filtered by title and abstract. A full-text assessment and review of the remaining studies was conducted. In the initial screening phase the following criteria have been utilized for filtering:

- Not population of interest
- Not entity of interest
- Relevant outcomes not reported
- Did not report real patient data, and instead reported Kaplan–Meier survival estimates without real patient survival

The two reviewers independently extracted data from the included articles using double-data extraction. Inconsistencies were resolved by consensus. In the case of disagreement, a third reviewer (CH) was consulted to reach a consensus.

### Eligibility criteria

All studies reporting metabolomic profiles in patients 18 years or older who underwent either EC, GEJ or GC surgery for any malignant condition besides sarcoma were considered eligible for inclusion. Studies aiming to find metabolomics characteristics and candidate metabolic biomarkers for diagnosis in EC, GEJ, and GC were included. The study used the PECOS acronym to define the inclusion and exclusion criteria: Population, Exposure, Comparison, Outcome, and Type of Study. Studies that satisfied the requirements established using the PECOS method were included.

PECOS inclusion criteria:

- P: adult patients (> 18 years of age) from any geographic location or gender
- E: patients with a confirmed diagnosis of UGC
- C: difference in the concentration of metabolites between UGC and healthy controls in preoperative plasma samples and intraoperative samples from the tumour and/or adjacent tissue
- O: dysregulation of metabolite concentrations between the predetermined study groups reported as either mean ± standard deviation, fold change concentration or log fold change concentration
- S: human-based observational studies (case–control, cohort, or cross-sectional) published since the inception of UGC metabolomics, i.e., January 2004 and January 2023, which used an untargeted or targeted metabolomic technique to quantify metabolite concentration

As well as metabolomics-specific inclusion criteria:

- studies investigating either human tissues or plasma
- the use of a detection platform including NMR, GC-MS, LC-MS, UPLC-MS/MS, HPLC-GC/MS-MS, or multiple platforms
- the names of differential metabolites available for extraction

When duplicated reports were published from the same population, the most recent or most complete publication was included. Studies with pregnant women, children and adolescents, those that addressed a disease other than UGC, review articles, guidelines, letters and editorials were excluded.

Exclusion criteria:

- Patients diagnosed with neoplasms other than UGC, either in past or present
- Patients suffering from any chronic systemic illness or on medication for the same
- components other than metabolites as biomarkers
- animal or cell-based studies
- non-observational study designs such as case reports, conference proceedings, and reviews
- Metabolites quantified other than in concentration, such as field of appearance, retention time, m/z ratio, etc.
- studies published before January 2004 or after January 2023
- genomics and proteomics research
- incomplete data

### Data extraction

For all selected articles, information on authors, publication year, sample type, analytical platform, sample size, and differentially distributed metabolites across comparison groups were independently extracted by two investigators (IB and SJ). In addition to individual metabolites, the two investigators independently reviewed findings on alterations in central metabolic pathways associated with upper GI cancers.

We used PubChem (https://pubchem.ncbi.nlm.nih.gov/ (accessed on December 11th 2023)) to search the metabolites needed to convert their units. The quartile and median or interquartile range (IQR) were converted into mean and standard deviation (SD). For median and quartile, we tested the skewness first and applied a new piecewise function based on the sample size.

Das Manuskript sollte eine klare Erläuterung der Bedingungen enthalten, unter denen Studien mit mehreren Versuchsgruppen im Verhältnis zu einer einzigen Kontrollgruppe zusammengeführt werden. Dazu gehören die Kriterien für die Einstufung der Gruppen als experimentell, die Art der Behandlungen oder die Bedingungen der Untergruppen (z. B. pathologisches Staging und Klassifizierung) sowie Strategien für den Umgang mit erheblicher Heterogenität.

When a study matches two or more experimental groups to one control group, the mean and standard deviation of the experimental group are combined. A qualitative analysis was performed to change the direction of metabolites by counting the frequency across the studies.

### Data synthesis and meta-analysis

Due to the lack of a standard procedure for meta-analysis of metabolomic data, in this study, we used SPSS software (PASW version 29.0, SPSS Inc. Chicago, IL, USA) as well as the machine learning algorithms Amanida and meta package in R (version: 4.2.2) (Egger et al. 1997). The Amanida and meta package enabled us to perform a meta-analysis of metabolomics data and combine the results of different studies addressing the same question in metabolomics profiles (Llambrich et al. 2022). A list of dysregulated metabolites was obtained from each study, considering the metabolite levels in healthy controls. Then, the input data were provided via text files containing the information of studies, including the identifiers (metabolite names), p values, fold-changes, study sizes, and references. Since different studies used different assessment methods, we used standardised mean difference (SMD) to assess the effect size. SMD was calculated using Cohen’s D. An effect size of 0.2 was considered to have a low effect, whereas 0.4 was a moderate effect, and 0.8 or more was a large effect. Alternatively, the effect measures used to determine the association between UGC and metabolite with the odds ratio (OR), hazard ratio (HR), incidence rate ratio (IRR) and summary relative risk (SRR) with respective 95% confidence intervals (CI). The significance level for this meta-analysis model was 0.05. We used Q statistic and I^2^ values to test for variability and assess the proportion of total variability due to heterogeneity, respectively. Heterogeneity was considered low, medium, and high, where I^2^ values of about 25%, 50% and 75% were present. The total amount of heterogeneity was estimated by heterogeneity variance (τ2) (Patti et al. 2012). A combination of weighted p-values, a modification of Fisher’s method, was used to evaluate the significance of a statistical result using the p-value. The fold change was logarithmically transformed (base 2) to reduce methodological bias, in which case the variation is more homogeneous, and the distribution of the sample mean matches a normal distribution. Log-transformed fold change values are averaged with weight by study size. Qualitative data analysis was performed using the vote-counting method. Vote counting involves the overall behaviour of metabolites per study. Votes are assigned as follows: a value of 1 for metabolites that are up-regulated, a value of − 1 for down-regulated, and 0 for no change in behaviour. The total votes for the composition are then added together. Herewith, our model incorporates both within-study and between-study variability (Higgins et al. 2002).

### Study quality assessment

According to the recommendation of the Cochrane Collaboration, the Newcastle–Ottawa scale (NOS) was used to determine the risk of bias, assess the overall reliability of the extracted data, and evaluate the methodological quality of the included studies, respectively (Stang 2010; Lin and Chu 2018). The NOS contains eight items (nine scores in total, where 9 indicates that the study meets all nine criteria for quality assessments and 0 indicates that the study does not meet any of the requirements), which fit into three categories: selection (four scores), comparability (two scores), and exposure of a case–control study or outcome of a cohort study (three scores). The articles were classified as “poor”, “fair”, “good”, or “excellent” when achieving scores of 0–3, > 3–6, > 6–8 or > 8–9, respectively (Lo et al. 2014a). Studies with less than five points were classified as having a high risk of bias.

In our study, the diagnostic accuracy and quality assessment of the included studies were reckoned by three reviewers (IB, SJ, and CH). We independently evaluated the quality of all selected articles using the QUADOMICS tool, an adaptation of the QUADAS-2 (Quality Assessment of Diagnostic Accuracy) tool (Lumbreras et al. 2008). To evaluate the quality of studies included in this systematic review, we addressed 16 selected questions for each to assess methodological subheadings used in metabolomic investigations. Each question is either answered “yes” or “no”. The methodologies of studies that achieved 12/16 or more on the QUADOMICS tool were classified as ‘high quality’, whereas those that scored 11/16 or lower were classified as ‘low quality’. For reference, each reviewer was provided with a copy of the QUADOMICS publication (Whiting et al. 2003) and the article evaluating QUADAS-2 and providing some modifications to the items (Whiting et al. 2006). All three researchers met to compare their observations and generate the consensus rating after ten articles had been reviewed, after 30, and finally after all 66; any disagreements were solved by discussion (Long, et al. 2020). The data set supporting the results of meta-analyses presented in this study is available in Supplementary Material.

## Results

The results were divided into five parts: (1) PRISMA literature research; (2) characteristics of the included studies for both qualitative (systematic review) and quantitative (meta-analysis) results; (3) systematic review results; (4) quality assurance results for the studies included; and (5) meta-analysis results.

### Literature search

**Fig. 1:**
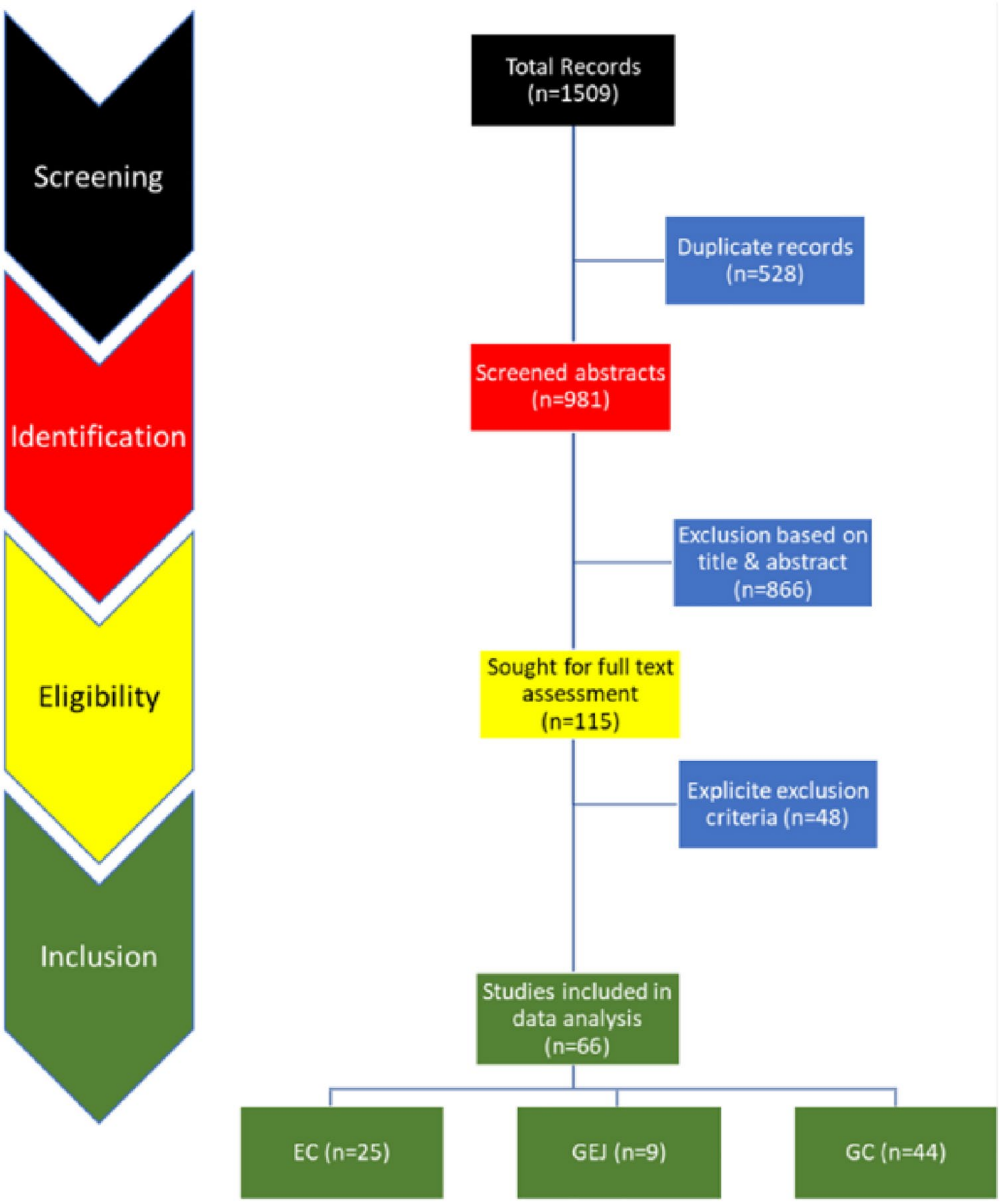
PRISMA flow diagram of search strategy and inclusion in the updated systematic review on metabolomics and upper GI cancers according to the PRISMA 2020 updated guidelines

The search returned a total of 1509 reports from Embase (571), Web of Science (109), MEDLINE (217) and PubMed (612). A total of 981 studies were identified after de-duplication. Screening by title and abstract led to the exclusion of 866 studies, leaving 115 studies that underwent full-text assessment (Fig. 1). We then excluded studies that were reviews, conference papers, book chapters, short surveys, notes, letters, or editorials. The final list of included investigations for the systematic review (qualitative synthesis) and for quantitative synthesis in the meta-analysis contained 66 studies, which investigated the metabolome of EC or/and GEJ or/and GC and met the criteria of minimum compounds per group and matched groups by age and sex, compound identification, ethics approval, and matrix sample storage.

### Characteristics of the included studies

The included records were published between 2004 and 2023, and all were cross-sectional, case–control, or cohort designs reported in English. Non-English articles were excluded from this review. Participants were from Europe, the United States, and Asia. Age and sex were well-matched in most of the studies. The studies were classified according to different sample types, including 41 blood samples and 29 tissue sample studies. All the included studies investigated the metabolome mainly using LC-MS, GC-MS, CE-MS techniques or NMR. The methodological and cohort characteristics of each study are shown in Table 1.

**Table 1:** Methodological and cohort information from selected studies' systematic review (qualitative analysis)

Sample type	Analytical platform	Ethics Approval	Cancer group	Sample size	Control group	Sample size	Storage	Ref
Plasma	LC–MS/MS	Yes	GC	10	Healthy subjects	31	– 80 °C	Li et al. (2023)
Tissue and Plasma	1H NMR	Yes	EC	70	Healthy subjects	70	– 80 °C	Ouyang et al. (2023)
Plasma	UPLC-TQ-MS	Yes	GC	150	Healthy subjects	151	– 80 °C	Yu et al. (2023)
Plasma	UPLC-MS	Yes	GC, GEJ	113	Healthy subjects	145	– 80 °C	Pan et al. (2022)
Plasma	LC–MS/MS	Yes	GC	72	Healthy subjects	29	– 80 °C	Matsumoto et al. (2023)
Plasma	LC–MS/MS	Yes	GC	69	Healthy subjects	146	– 80 °C	Li et al. (2022)
Plasma	GC–MS	Yes	EC, GEJ	121	Healthy subjects	30	– 80 °C	Yang et al. (2022a)
Dried blood	LC–MS/MS	Yes	GC	166	Healthy subjects	183	– 80 °C	Wu et al. (2022)
Plasma	LC–MS/MS	Yes	GC	112	Healthy subjects	112	– 80 °C	Yuan et al. (2022)
Tissue	UHPLC-MS	Yes	EC	60	Healthy subjects	15	– 80 °C	Yang et al. (2022b)
Tissue	LC–MS/MS	Yes	GC	28	Healthy subjects	28	– 80 °C	Wang, et al. (2021)
Plasma	LC–MS/MS	Yes	GC, GEJ	30	Healthy subjects	170	– 80 °C	Huang et al. (2021)
Plasma	LC–MS/MS	Yes	GC	250	Healthy subjects	250	– 80 °C	Shu et al. (2021)
Tissue and Plasma	1H NMR	Yes	EC	50	Healthy subjects	50	– 80 °C	Ye et al. (2021)
Plasma	UHPLC-Q-TOF/MS	Yes	GC	51	Healthy subjects	40	– 80 °C	Yu et al. (2021)
Plasma	NMR	Yes	GC	103	Healthy subjects	100	– 80 °C	Kwon, et al. (2020)
Tissue	LC–MS/MS	Yes	GC	62	Healthy subjects	62	– 80 °C	Pan et al. (2020)
Tissue	CE-TOF/MS	Yes	GC	140	Healthy subjects	140	– 80 °C	Kaji et al. (2020)
Plasma	LC–MS/MS	Yes	GC	47	Healthy subjects	47	– 80 °C	Wang et al. 2020)
Plasma	UHPLC–MS/MS	Yes	GC, GEJ	20	Healthy subjects	20	– 80 °C	Lee et al. (2019a)
Plasma	LC–MS/MS	Yes	GC	71	Healthy subjects	54	– 80 °C	Corona, et al. 2018)
Tissue	CE–TOF/MS	Yes	EC	35	Healthy subjects	35	– 80 °C	Tokunaga et al. (2018)
Plasma	LC–MS/MS	Yes	EC	84	Healthy subjects	82	– 80 °C	Jing et al. (2018)
Plasma	LC–MS/MS	Yes	EC	34	Healthy subjects	32	– 80 °C	Ma et al. (2018)
Plasma	LC–MS/MS	Yes	GC	20	Healthy subjects	60	– 80 °C	Lario et al. 2017)
Tissue	LC–MS/MS	Yes	EC	40	Healthy subjects	40	– 80 °C	Zhang et al. (2017)
Plasma	LC–MS/MS	Yes	EC	40	Healthy subjects	27	– 80 °C	Cheng et al. (2017)
Tissue	GC/TOF–MS	Yes	EC	43	Healthy subjects	40	– 80 °C	Zhu et al. (2017)
Tissue	1H NMR	Yes	EC	46	Healthy subjects	75	– 80 °C	Reed et al. (2017)
Plasma	HPLC/ESI/Q-TOF/MS	Yes	GC	125	Healthy subjects	38	– 80 °C	Wang et al. (2017)
Plasma	LC–MS/MS	Yes	GC	35	Healthy subjects	17	– 80 °C	Choi et al. (2016)
Tissue	1H NMR	Yes	GC	125	Healthy subjects	54	– 80 °C	Wang et al. (2016)
Plasma	1H-NMR	Yes	GC	43	Healthy subjects	80	– 80 °C	Chan et al. (2016)
Plasma	UPLC–TOF/MS	Yes	GC	33	Healthy subjects	110	– 80 °C	Kuligowski et al. (2016)
Plasma	LC–MS/MS	Yes	EC	62	Healthy subjects	62	– 80 °C	Xu et al. (2016)
Plasma	LC–MS/MS	Yes	GC	13	Healthy subjects	9	– 80 °C	Liang et al. (2015)
Plasma	LC–MS/MS	Yes	EC	40	Healthy subjects	10	– 80 °C	Mir et al. 2015)
Tissue	1H NMR	Yes	GC, GEJ	80	Healthy subjects	80	´ – 80 °C	Jung et al. 2014)
Plasma	HPLC/ESI–MS/MS	Yes	GC	49	Healthy subjects	40	– 80 °C	Lo et al. (2014b)
Plasma	MRB–CE–MS	Yes	GC	26	Healthy subjects	14	– 80 °C	Chen et al. (2014)
Tissue	GC–MS	Yes	GC	45	Healthy subjects	45	– 80 °C	Hur et al. (2014)
Tissue	MALDI MS	Yes	GC	12	Healthy subjects	12	– 80 °C	Kwon, et al. (2014)
Tissue	1H NMR	Yes	EC	17	Healthy subjects	14	– 80 °C	Yang et al. (2013)
Plasma	1H NMR and UHPLC	Yes	EC	25	Healthy subjects	25	– 80 °C	Zhang et al. (2013)
Plasma	UPLC/TOF/MS	Yes	EC	53	Healthy subjects	53	– 80 °C	Liu et al. (2013)
Tissue	1H NMR	Yes	EC	89	Healthy subjects	26	– 80 °C	Wang et al. 2013)
Plasma	GC–MS	Yes	GC, EC, GEJ	26	Healthy subjects	12	– 80 °C	Ikeda et al. (2012)
Tissue	SIFT-MS	Yes	GC, EC, GEJ	19	Healthy subjects	20	– 80 °C	Kumar et al. (2012)
Plasma	GC–MS	Yes	GC	30	Healthy subjects	30	– 80 °C	Song et al. (2012)
Plasma and tissue	GC/TOFMS	Yes	GC	32	Healthy subjects	20	– 80 °C	Aa et al. (2012)
Plasma	NMR	Yes	EC	108	Healthy subjects	40	– 80 °C	Hasim et al. (2012)
Plasma	LC–MS and NMR	Yes	EC	67	Healthy subjects	46	– 80 °C	Zhang et al. (2012)
Plasma	1H NMR	Yes	EC, GEJ	44	Healthy subjects	106	– 80 °C	Davis et al. (2012)
Tissue	HPLC–MS	Yes	GC	33	Healthy subjects	68	– 80 °C	Deng et al. (2011)
Plasma	GC/TOF–MS	Yes	GC	22	Healthy subjects	57	– 80 °C	Yu et al. (2011)
Plasma	NMR	Yes	EC	68	Healthy subjects	50	– 80 °C	Zhang, et al. (2011)
Tissue	GC–MS	Yes	GC	18	Healthy subjects	18	– 80 °C	Wu et al. 2010)
Tissue	NMR	Yes	EC	52	Healthy subjects	35	– 80 °C	Yakoub et al. 2010)
Tissue	GC–MS	Yes	GC, GEJ	65	Healthy subjects	65	– 80 °C	Cai et al. (2010)
Plasma	HPLC/TQ/MS	Yes	EC	14	Healthy subjects	12	– 80 °C	Djukovic et al. (2010)
Tissue	CE–MS	Yes	GC	12	Healthy subjects	12	– 80 °C	Hirayama et al. (2009)
Tissue	SPME-GC/MS	Yes	GC	5	Healthy subjects	5	– 80 °C	Buszewski et al. (2008)
Tissue	HR-MAS MRS	Yes	GC	5	Healthy subjects	22	– 80 °C	Calabrese et al. (2008)
Tissue	SPME-GC/MS	Yes	GC	3	Healthy subjects	13	– 80 °C	Ligor et al. (2007)
Tissue	HR-MAS NMR	Yes	GC	5	Healthy subjects	11	– 80 °C	Tugnoli et al. (2006)
Tissue	1H MRS	Yes	GC	13	Healthy subjects	22	– 80 °C	Mun et al. (2004)

*MS/MS* tandem mass spectrometry, *HPLC* high-performance liquid chromatography, *CE* capillary electrophoresis, *UPLC* ultra-performance liquid chromatography

### Systematic review

In 66 studies a total of 169 metabolites; 142 in blood samples, and 65 in tissue samples were identified to significantly differentiate patients with UGC from healthy controls. These investigations comprised 7267 samples in total, of whom 3650 were samples from a UGC and 3617 were healthy controls. 12 of the included studies were conducted in Europe, 38 in Asia, and 17 in America. Each study reported the compound name translated to InChIKey with the chemical translation service (http://cts.fiehnlab.ucdavis.edu/batch (accessed on December 11th 2023). These results were compared to match the compound identifiers between articles, as only some authors reported the same compound in the same manner. If the CTS service did not find a compound, a manual search was performed at PubChem (https://pubchem.ncbi.nlm.nih.gov/ (accessed on December 12th 2023)). In most studies, investigators used MS coupled with different chromatography techniques to generate the metabolomic data (*n* = 51). In 14 studies, an NMR platform was used, and two used a combination of MS and NMR platforms. The monoisotopic mass from all compounds ranged from 31.06 g/mol for methylamine with only one carbon to 859.74 g/mol for phosphatidylcholine (42:0) with 50 carbons. The compounds most repeated in the literature were branched-chain amino acids (BCAA; isoleucine, leucine, and valine) identified by 8 studies. The most repeated compounds in the literature after BCAA were phosphatidylcholines, eicosanoids, and carbohydrates from the tricarboxylic acid cycle. Based on the sample type classification, 97 metabolites showed increased concentrations, 58 showed decreased concentrations, and 14 showed inconsistent concentrations in blood and tissue samples. The details of the qualitative synthesis are given in Supplementary Table 1.

### Quality assurance

**Fig. 2:**
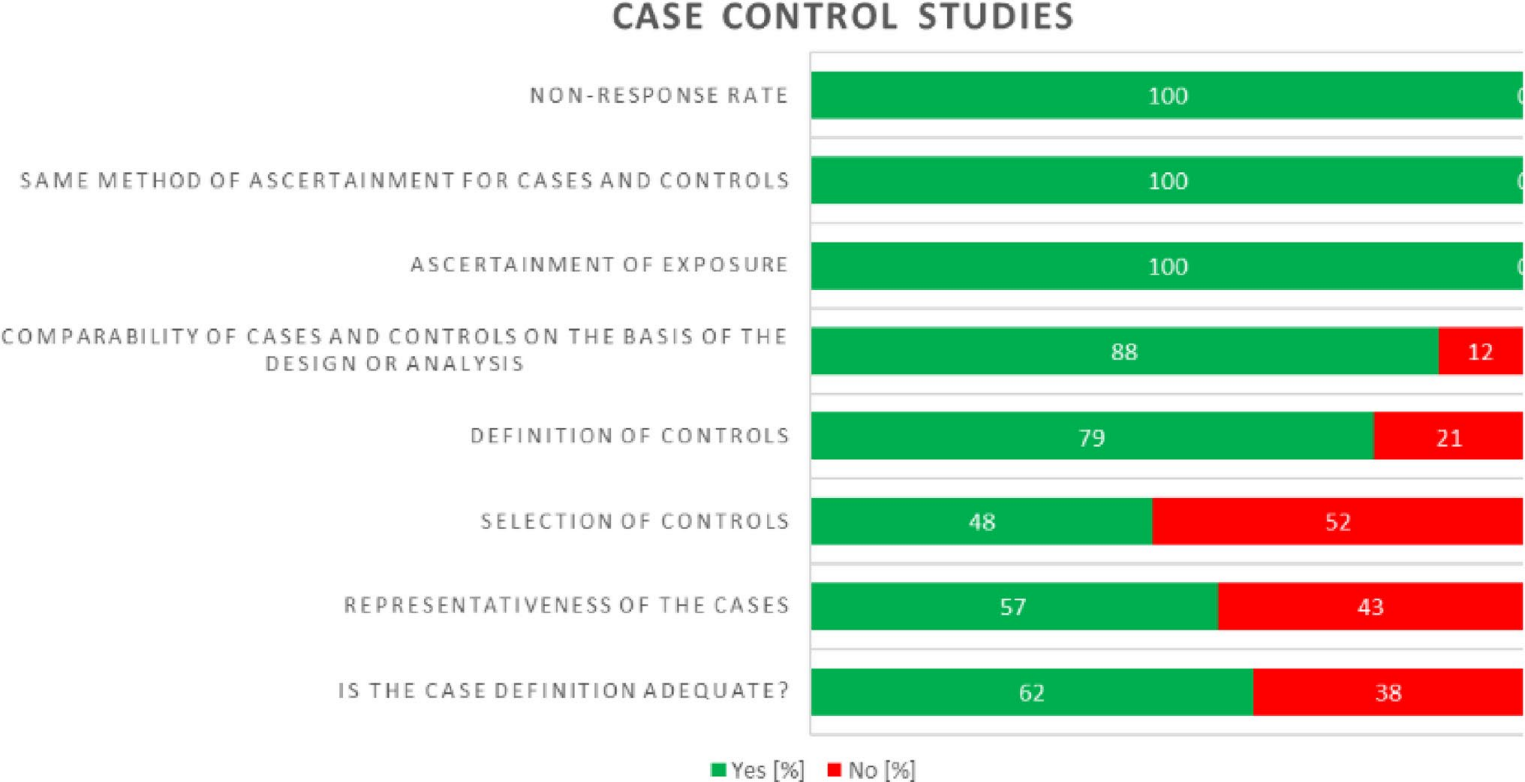
Summary of quality assessment of included studies using the NOS for case–control studies. The proportion of studies satisfying the criteria is plotted on a scale of 100%

**Fig. 3:**
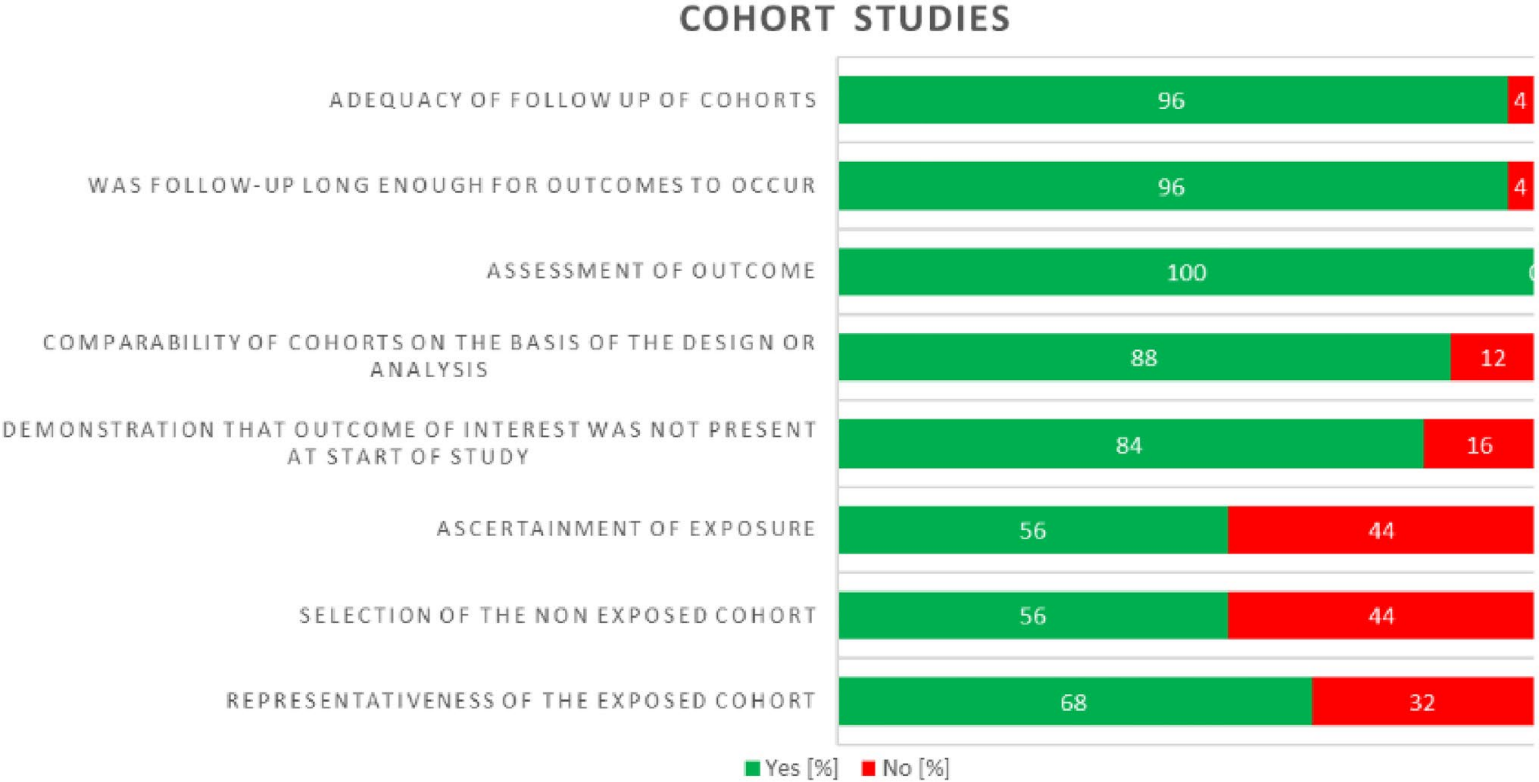
Summary of quality assessment of included studies using the NOS for cohort studies. The proportion of studies satisfying the criteria is plotted on a scale of 100%

**Fig. 4:**
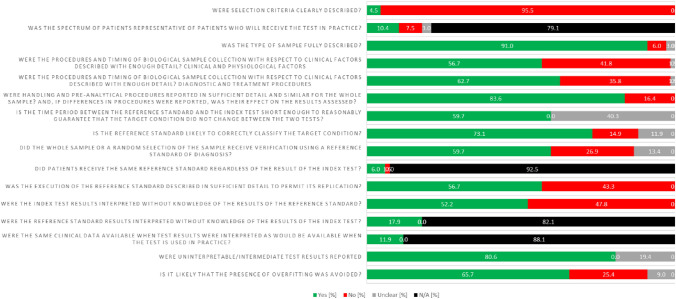
Summary of quality assessment of included studies based on the QUADOMICS tool. The proportion of studies satisfying the criteria is plotted on a scale of 100%

Quality assurance of the included studies was performed, using the NOS and the QUADOMICS tool for evaluation. The quality assurance results are shown in Figs. 2, 3, and 4. Variables were based on the experimental methodology.

In case–control studies, the most reported domains were in “*Same method of ascertainment for cases and controls*”, *“Ascertainment of exposure”*, and *“non-Response rate”*, with more than 90% of studies reporting complete information. On the other hand, the least reported domains were in *“Selection of Controls”* and *“Representativeness of the cases”*, where less than 60% of the studies disclosed complete information.

In contrast to case–control studies, cohort studies reported the most domains in “*Adequacy of follow up of cohorts*”, *“Was follow-up long enough for outcomes to occur”*, and *“Assessment of outcome”*, with more than 90% of studies reporting complete information. The least reported domains were in *“Selection of the non-exposed cohort”* and *“Ascertainment of exposure”*, where less than 60% of the studies disclosed complete information.

As a summary, of the 66 cross-sectional studies, 54 attained a NOS score of 6 or higher (“good” or “excellent”; thus, moderate to low risk of bias). We deemed 12 studies to have a relatively high risk of bias (NOS score: < 6) because of their small and unjustified sample size and lack of statistical adjustments for confounding factors. However, excluding this study did not alter our risk estimate.

According to QUADOMICS, we conducted a quality assessment process (see the score in Fig. 4). The general characteristics of the selected studies and methodological quality assessment were independently checked by IB, SJ and CH.

The quality assessment process following the QUADOMICS tool is summarised in Supplementary Table 1. 6 of the 66 studies were classified as ‘low quality’, fulfilling fewer than 12 of the 16 criteria. The most common problems were *not reporting the justification*, *sample size calculation*, and the *number of non-respondents*. Although the funnel plot was asymmetric on visual inspection, and a potential publication bias was also found in Egger’s test (p = 0.006, Supplementary Table 1), the effect size was not significantly changed after the trim-and-fill (OR = 1.12, 95%CI [1.01, 1.25], p < 0.05, number of trim and fill = 4), thus indicating that the publication bias had little influence on the results. The complete appraisal of the methodological quality of the articles is described in Supplementary Table 1.

### Meta-analysis

**Fig. 5:**
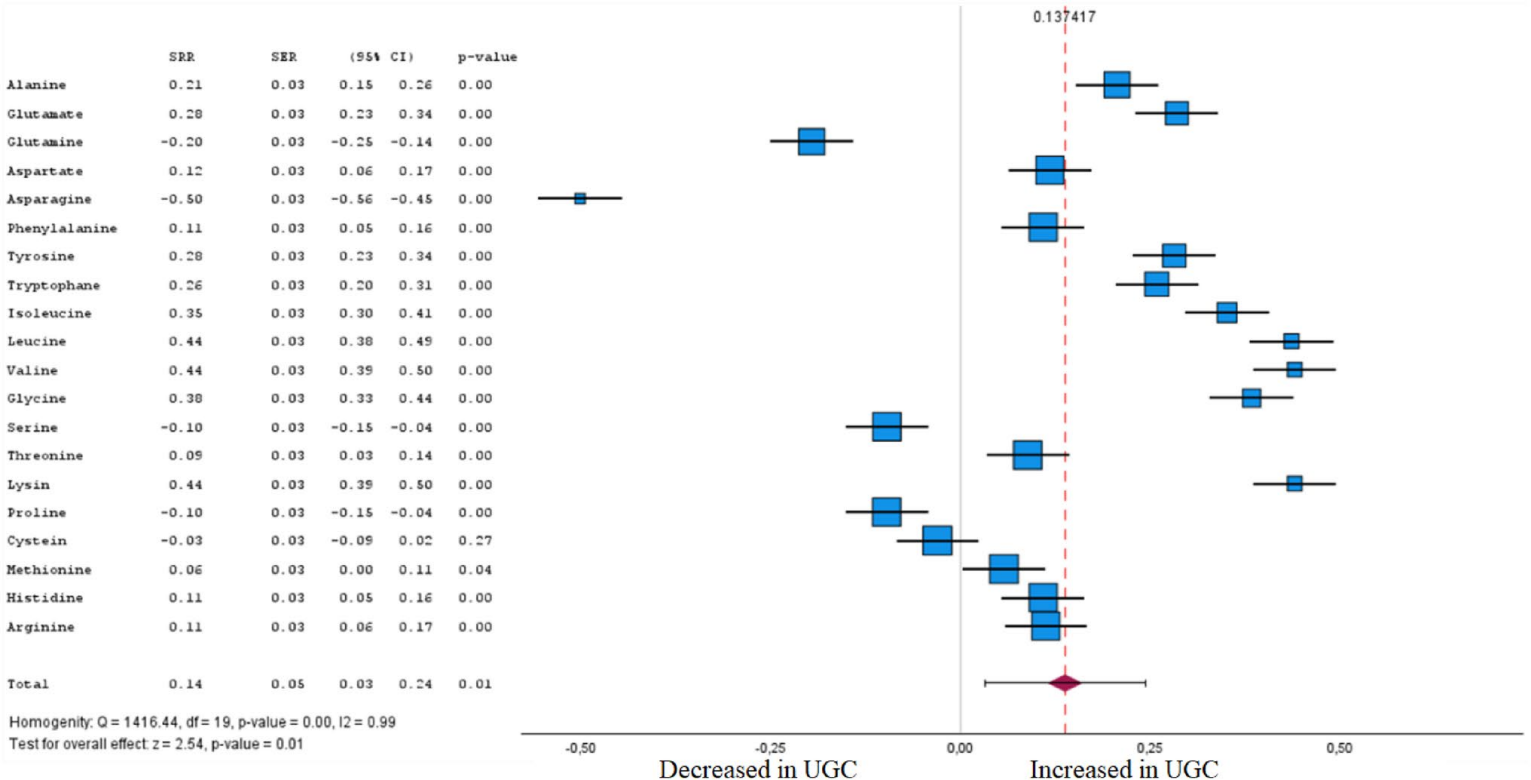
Forest plot of pooled estimates of UGC associated per study-specific difference in each amino acid from case–control and cohort studies. Overall estimates were obtained from forest plots and random-effects meta-analysis of studies evaluating metabolites and incidence of UGC. Estimates were derived from the most fully adjusted model in each included analysis. Closed squares and horizontal bars represent the Summary relative risk (SRR) with corresponding 95% CIs

**Fig. 6:**
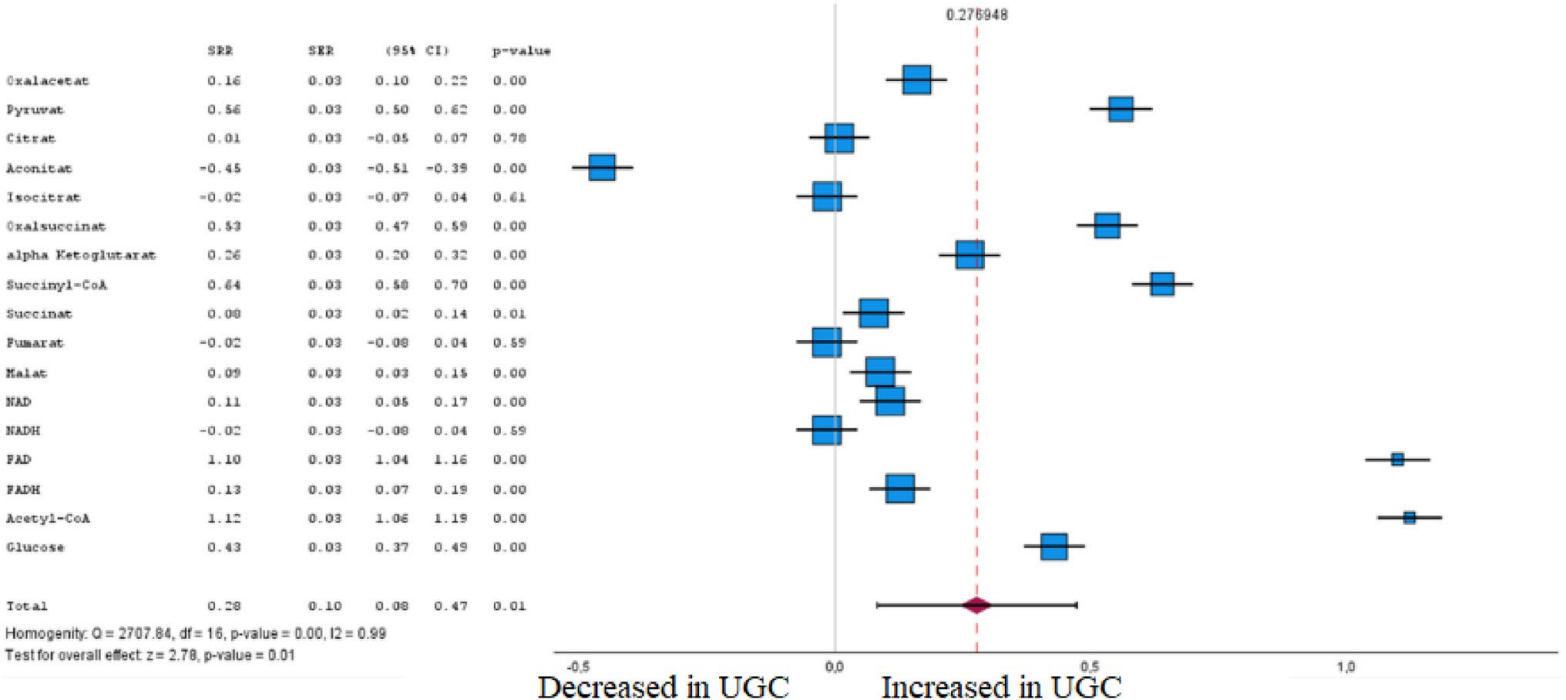
Forest plot of pooled estimates of UGC associated per study-specific difference in each metabolite from citrate cycle from case–control and cohort studies. Overall estimates were obtained from forest plots and random-effects meta-analysis of studies evaluating metabolites and incidence of UGC. Estimates were derived from the most fully adjusted model in each included analysis. Closed squares and horizontal bars represent the Summary relative risk (SRR) with corresponding 95% CIs

**Fig. 7:**
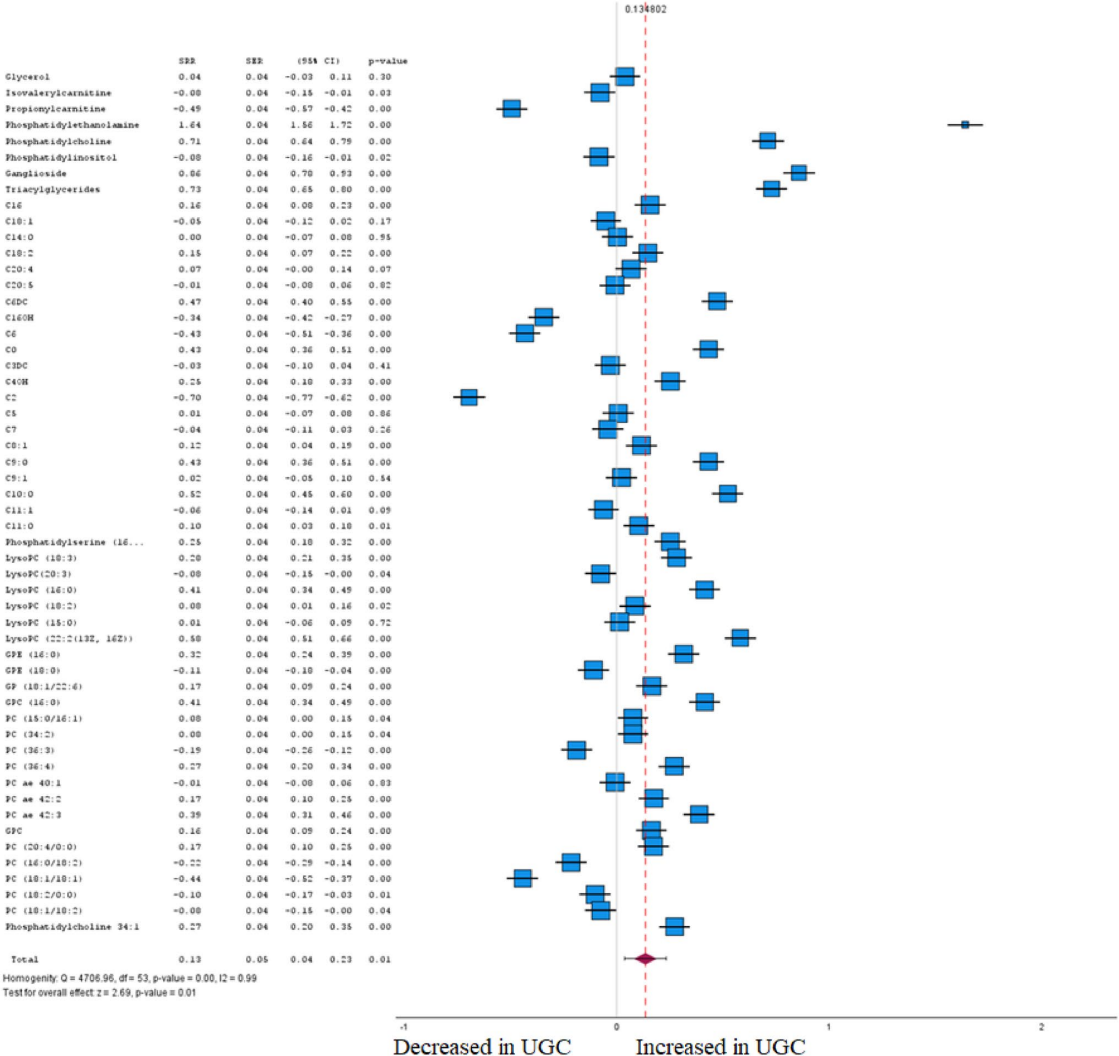
Forest plot of pooled estimates of UGC associated per study-specific difference in each metabolite from case–control and cohort studies. Overall estimates were obtained from forest plots and random-effects meta-analysis of studies evaluating metabolites and incidence of UGC. Estimates were derived from the most fully adjusted model in each included analysis. Closed squares and horizontal bars represent the Summary relative risk (SRR) with corresponding 95% CIs

**Fig. 8:**
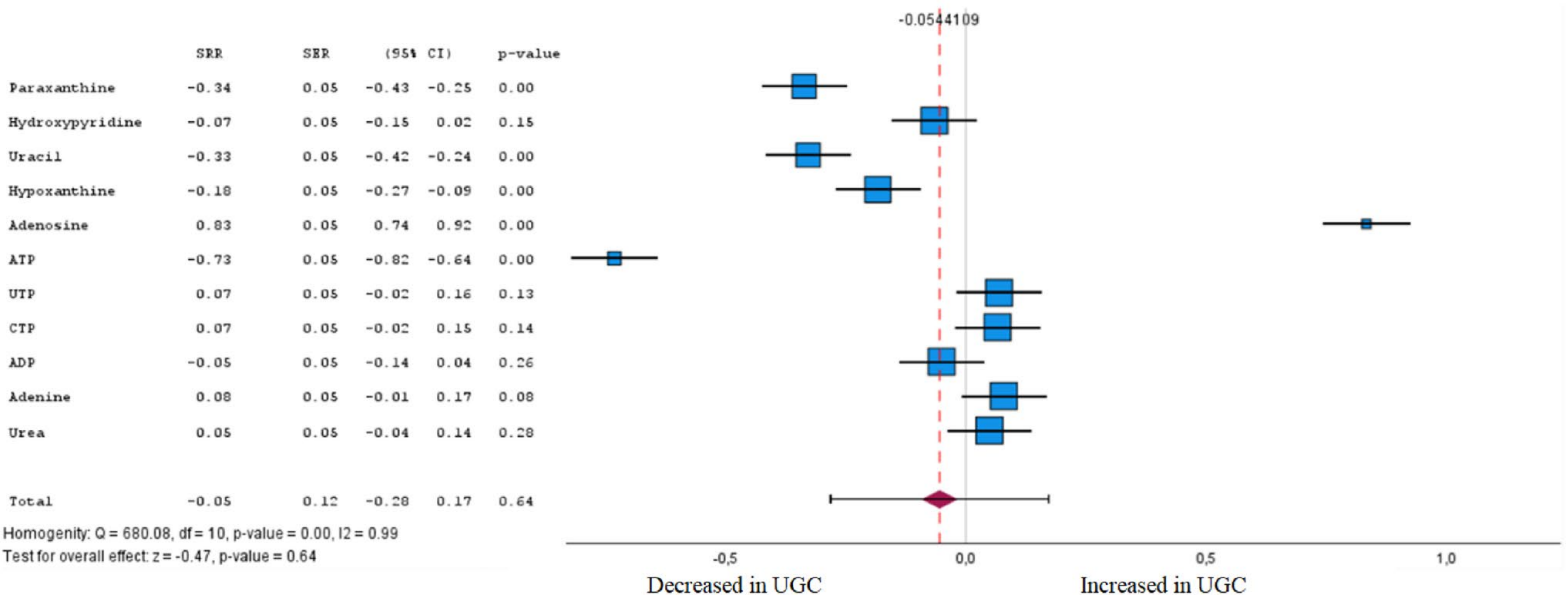
Forest plot of pooled estimates of UGC associated per study-specific difference in each metabolite from purine and pyrimidine metabolism from case–control and cohort studies. Overall estimates were obtained from forest plots and random-effects meta-analysis of studies evaluating metabolites and incidence of UGC. Estimates were derived from the most fully adjusted model in each included analysis. Closed squares and horizontal bars represent the Summary relative risk (SRR) with corresponding 95% CIs

**Fig. 9:**
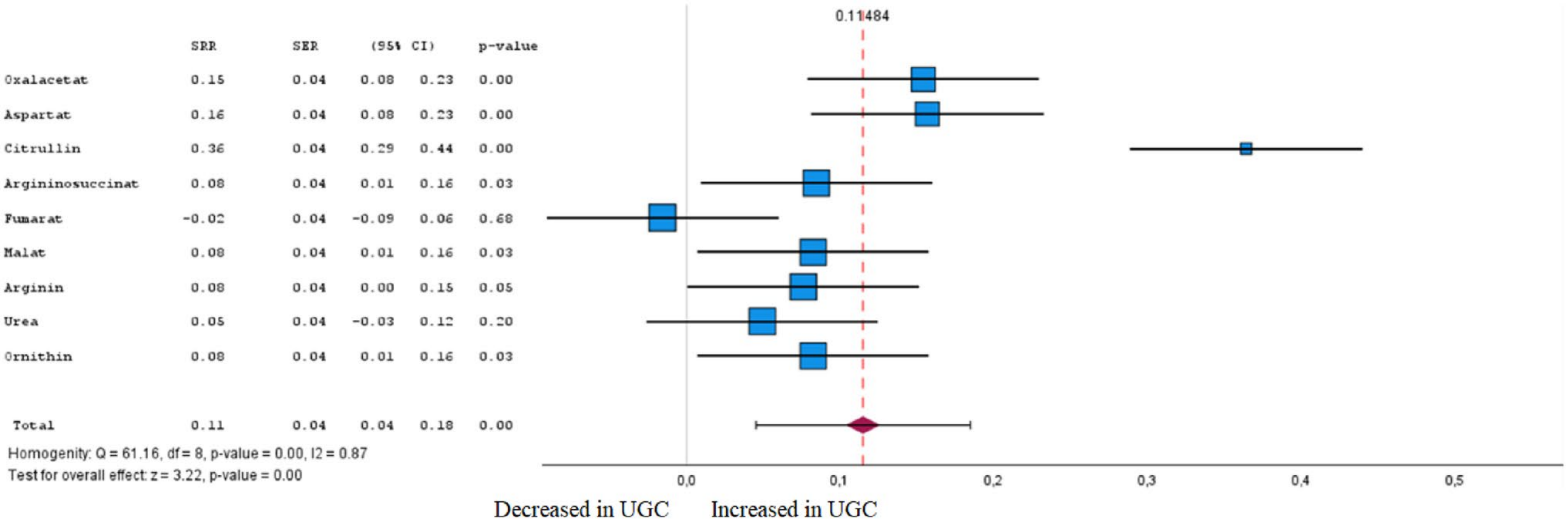
Forest plot of pooled estimates of UGC associated per study-specific difference in each metabolite from urea cycle from case–control and cohort studies. Overall estimates were obtained from forest plots and random-effects meta-analysis of studies evaluating metabolites and incidence of UGC. Estimates were derived from the most fully adjusted model in each included analysis. Closed squares and horizontal bars represent the Summary relative risk (SRR) with corresponding 95% CIs

**Fig. 10:**
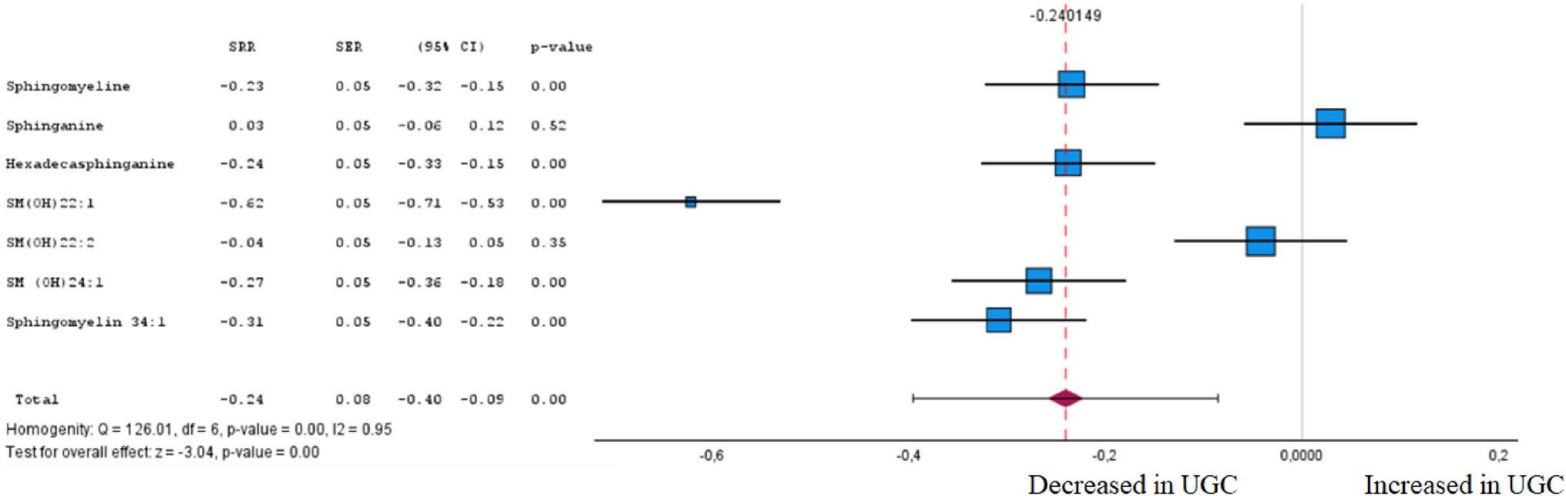
Forest plot of pooled estimates of UGC associated per study-specific difference in each sphingolipid from case–control and cohort studies. Overall estimates were obtained from forest plots and random-effects meta-analysis of studies evaluating metabolites and incidence of UGC. Estimates were derived from the most fully adjusted model in each included analysis. Closed squares and horizontal bars represent the Summary relative risk (SRR) with corresponding 95% CIs

**Fig. 11:**
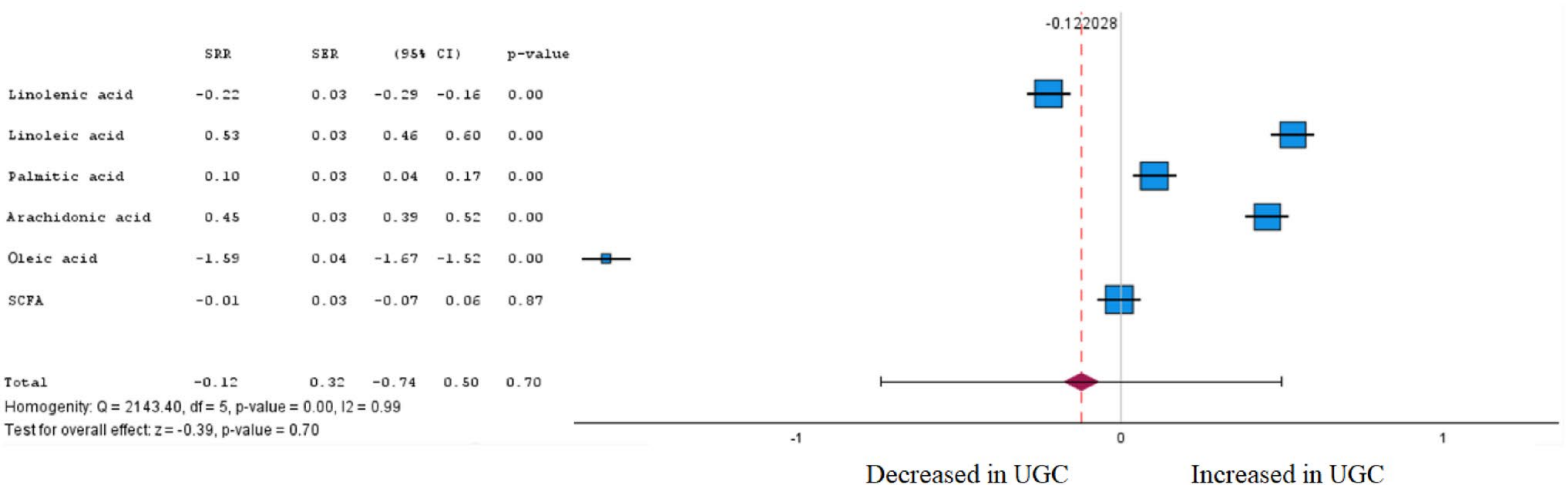
Forest plot of pooled estimates of UGC associated per study-specific difference in each fatty acid from case–control and cohort studies. Overall estimates were obtained from forest plots and random-effects meta-analysis of studies evaluating metabolites and incidence of UGC. Estimates were derived from the most fully adjusted model in each included analysis. Closed squares and horizontal bars represent the Summary relative risk (SRR) with corresponding 95% CIs

**Fig. 12:**
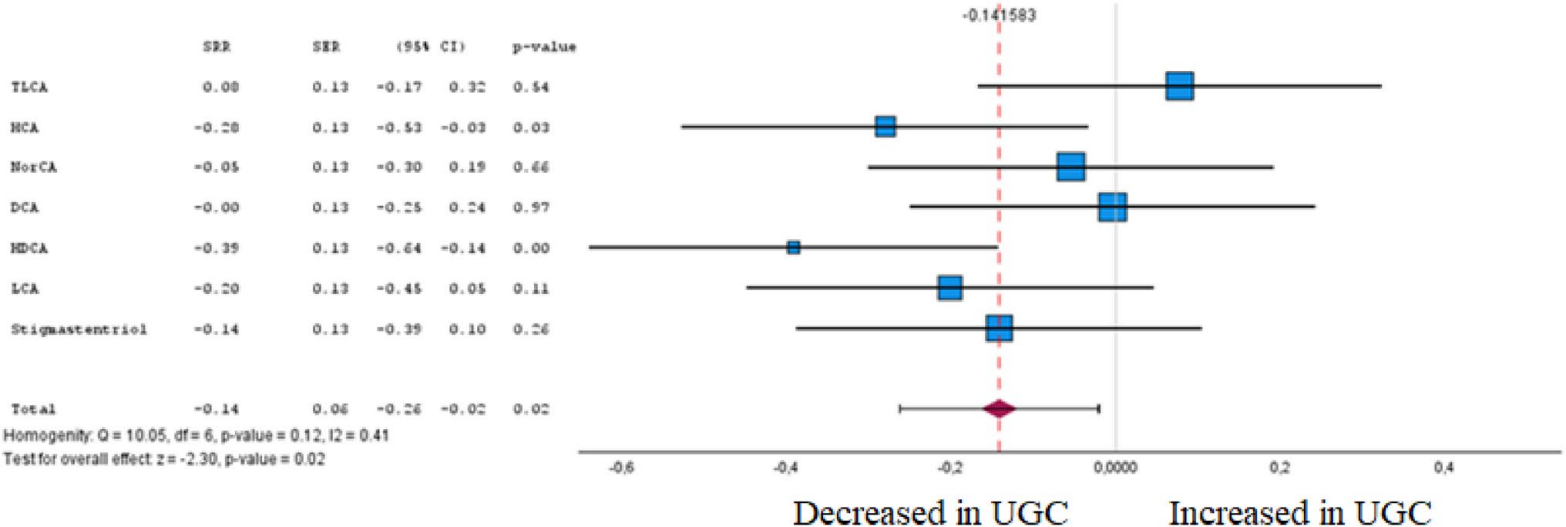
Forest plot of pooled estimates of UGC associated per study-specific difference in each bile acid from case–control and cohort studies. Overall estimates were obtained from forest plots and random-effects meta-analysis of studies evaluating metabolites and incidence of UGC. Estimates were derived from the most fully adjusted model in each included analysis. Closed squares and horizontal bars represent the Summary relative risk (SRR) with corresponding 95% Cis

**Fig. 13:**
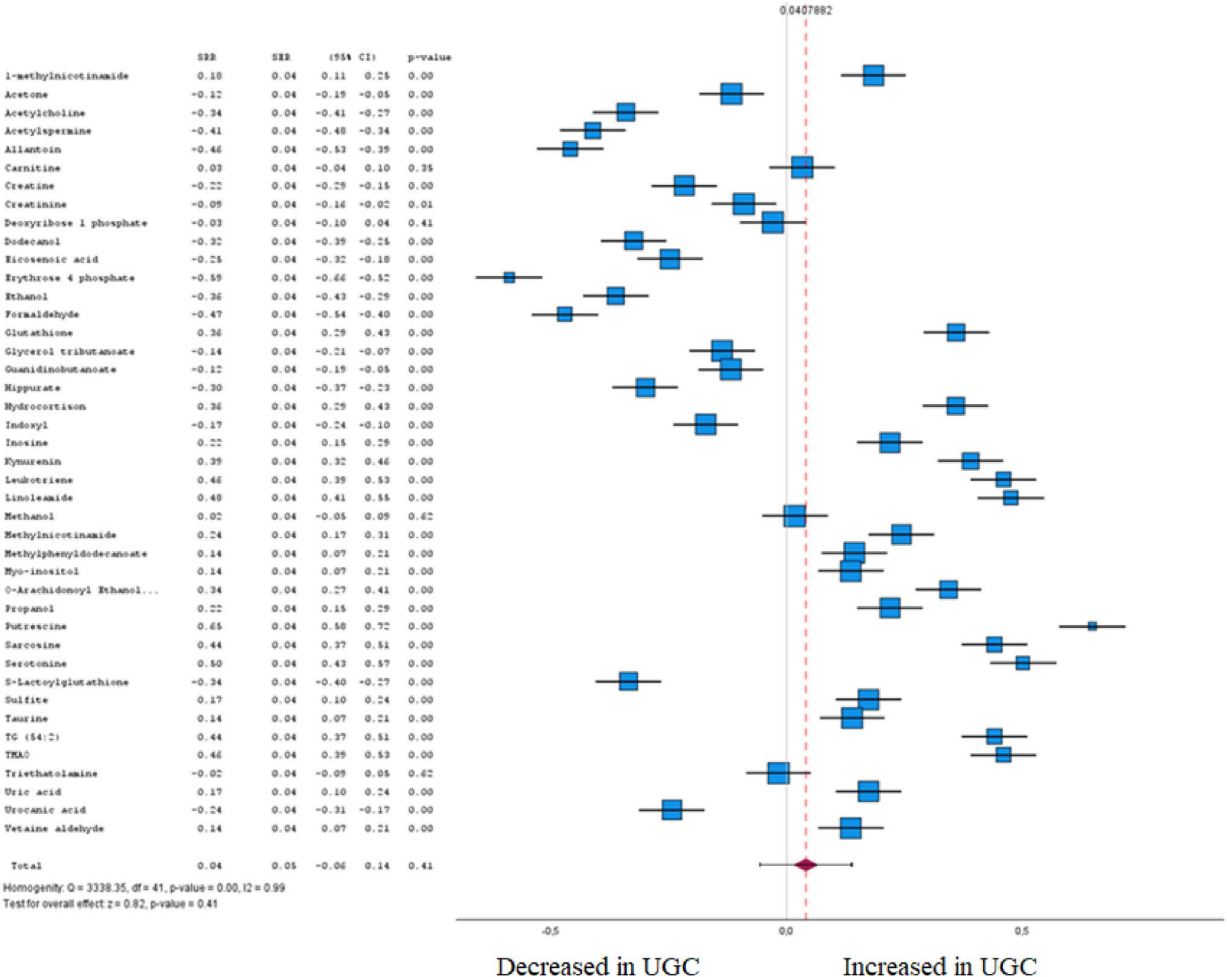
Forest plot of pooled estimates of UGC associated per study-specific difference in each metabolite from case–control and cohort studies. Overall estimates were obtained from forest plots and random-effects meta-analysis of studies evaluating metabolites and incidence of UGC. Estimates were derived from the most fully adjusted model in each included analysis. Closed squares and horizontal bars represent the Summary relative risk (SRR) with corresponding 95% CIs

Statistical analysis was performed for those groups of studies that reported the p values and fold-changes for significant compounds. Overall, we extracted the relative risk estimates for meta-analysis from a total of 169 metabolites from 66 studies. Among these, 155 metabolites were found to be significantly altered in healthy and tumour tissue by meta-analysis, while 14 were not. The forest plots for different metabolites between UGC and healthy controls and the meta-analysis results for different metabolites among UGC and controls are shown in Figs. 5, 6, 7, 8, 9, 10, 11, 12, 13 clustered in their respective biochemical classes.

### Amino acids

We performed a meta-analysis for 20 proteinogenic amino acids (Fig. 5 and Supplementary Table 1); 19 showed altered concentrations between patients with UGC and healthy controls (FDR-corrected P < 0.05) in tumour tissue and plasma. In accordance with Cohen's effect size, five metabolites were identified in the meta-analysis that exhibited both a significant difference between the cohorts and at least a moderate effect. Among these are higher circulating levels of branched-chain amino acids (BCAA, isoleucine, leucine, valine) (SRR 0.44 for leucine [95% CI 0.38–0.49], 0.44 for valine [95% CI 0.39–0.50], and 0.35 for isoleucine [95% CI 0.30–0.41]), were associated (FDR-corrected P < 0.05) with UGC. Also, lysine was associated with UGC (SRR 0.44 [95% CI 0.39–0.50]). An inverse association was observed for asparagine and UGC (SRR -0.50 [95% CI – 0.56 to – 0.45]). Decreased concentrations of glutamine were also linked to UGC (SRR -0.20 [95% CI – 0.25 to – 0.14]).

### Carbohydrates and tricarboxylic acid cycle

In total, 17 carbohydrates and tricarboxylic acid cycle (TCA) metabolites in tumour tissue and plasma were included in the meta-analysis (Fig. 6 and Supplementary Table 1), and 13 of them were associated with UGC (FDR-corrected P < 0.05). In accordance with Cohen’s effect size, three metabolites were identified in the meta-analysis that exhibited both a significant difference between the cohorts and at least a moderate effect. For these metabolites, we found that notably higher flavin adenine dinucleotide (FAD) (SRR 1.10 [95% CI 1.04–1.16]) and Acetyl-CoA (SRR 1.12 [95% CI 1.06–1.19]) levels were associated with UGC. One metabolite from the citrate cycle, aconitate (SRR – 0.45 [95% CI – 0.51 to – 0.39]), was inversely associated with UGC.

### Glycerophospholipids

In the biochemically heterogenous group of 54 glycerophospholipids and their direct derivates, we found 43 of them to be significantly associated with UGC (FDR-corrected P < 0.05) in tumour tissue and plasma (Fig. 7 and Supplementary Table 1). In accordance with Cohen's effect size, several metabolites were identified in the meta-analysis that exhibited both a significant difference between the cohorts and at least a moderate effect. Within the group of acylcarnitines, especially short- to medium-chain acyls, e.g., C2 (SRR -0.70 [95% CI – 0.77 to – 0.62]), C6 (SRR – 0.43 [95% CI – 0.51 to – 0.36]), and C16-OH (SRR -0.34 [95% CI – 0.42 to – 0.27]) resulted in having an inverse association with UGC. No metabolites resulted in a definitive association with UGC in the biochemical group of lysophosphatidylcholines. However, long-chained phosphatidylcholines, exemplary PC (18:1/18:1) (SRR – 0.44 [95% CI – 0.52 to – 0.37]) showed an inverse association with UGC. In Summary, these results indicate that the constellation of phosphatidylcholines, the main constituents of the cell membrane, distinctly differs between healthy cells of the upper gastrointestinal tract and tumour tissue from patients with UGC.

### Purine and pyrimidine metabolism

Regarding purines and pyrimidines, essential metabolites of nucleotide metabolism, our meta-analysis resulted in 11 compounds associated with UGC in tumour tissue and plasma (Fig. 8 and Supplementary Table 1). In accordance with Cohen's effect size, five metabolites were identified in the meta-analysis that exhibited both a significant difference between the cohorts and at least a moderate effect. Higher levels of one were associated (FDR-corrected P < 0.05) with UGC. In particular, ATP (SRR – 0.73 [95% CI – 0.82 to – 0.64]) was inversely associated with UGC. However, adenosine, a nucleoside composed of adenine and D-ribose, was associated with UGC (SRR 0.83 [95% CI 0.74–0.92]).

### Urea cycle

In the meta-analysis for metabolites involved in the urea cycle, six out of nine metabolites were significantly associated (FDR-corrected P < 0.05) with UGC in tumour tissue and plasma (Fig. 9 and Supplementary Table 1). In accordance with Cohen's effect size, six metabolites were identified in the meta-analysis that exhibited both a significant difference between the cohorts and at least a moderate effect. No definitive inverse associations with UGC were found for metabolites and derivates from the urea cycle. However, all six metabolites showed a direct association with UGC. Especially citrulline, also produced from arginine as a by-product, was associated with UGC (SRR 0.36 [95% CI 0.29–0.44]).

### Sphingolipids

Analysing sphingolipids (SM), we found seven SMs in tumour tissue and plasma, of which one was significantly (FDR corrected P < 0.05) associated with UGC and four were inversely associated with UGC (Fig. 10 and Supplementary Table 1). In accordance with Cohen's effect size, sphinganine was identified in the meta-analysis to exhibit both a significant difference between the cohorts and at least a moderate effect. The inverse association with UGC was observed for sphinganine (SRR 0.03 [95% CI – 0.06 to 0.12]). Among the remaining sphingolipids especially SM (OH) 22:1, a sphingolipid in the membranous myelin sheath which surrounds some nerve cell axons, inversely associated with UGC (SRR -0.62 [95% CI – 0.71 to – 0.53]).

### Non-esterified fatty acids

Among six non-esterified fatty acids included in the meta-analysis (Fig. 11 and Supplementary Table 1), two were associated with UGC, and three were inversely associated with UGC (FDR corrected P < 0.05) in tumour tissue and plasma. In accordance with Cohen’s effect size, two metabolites were identified in the meta-analysis that exhibited both a significant difference between the cohorts and at least a moderate effect. Linoleic acid (SRR 0.53 [95% CI 0.46–0.60]), a doubly unsaturated essential fatty acid, also known as an omega-6 fatty acid, was the fatty acid found to be associated the most with UGC, and linolenic acid an omega-3 fatty acids were inversely associated with UGC (SRR -0.22 [95% CI – 0.29 to – 0.16]).

### Bile acid

Among seven bile acids included in the meta-analysis (Fig. 12 and Supplementary Table 1), only two levels were associated (FDR-corrected P < 0.05) with UGC in tumour tissue and plasma. In accordance with Cohen's effect size, two metabolites were identified in the meta-analysis that exhibited both a significant difference between the cohorts and at least a moderate effect. Both of them, hyocholic acid (HCA) (SRR – 0.28 [95% CI – 0.53 to – 0.03]) and hyodeoxycholic acid (HDCA) (SRR -0.39 [95% CI – 0.64– – 0.14]) resulted in inverse association with UGC.

### Biogenic amines and related

Among 42 biogenic amines and related included in the meta-analysis (Fig. 13 and Supplementary Table 1), 38 levels were associated with a UGC in tumour tissue and plasma (FDR-corrected P < 0.05). In accordance with Cohen's effect size, several metabolites were identified in the meta-analysis that exhibited both a significant difference between the cohorts and at least a moderate effect. Within this biochemically heterogeneous group of metabolites, erythrose-4-phosphate (SRR – 0.59 [95% CI – 0.66 to – 0.52]), a monosaccharide comprising a phosphate group linked to the carbohydrate unit as well as putrescine a polyamine produced by the breakdown of amino acids were associated with UGC (SRR 0.65 [95% CI 0.58–0.72]).

Interestingly, Trimethylamine N-oxide (TMAO) (SRR 0.46 [95% CI 0.39–0.53]), a metabolite frequently reported to be associated with esophageal cancer, cancer of the gastroesophageal junction and gastric cancer, was associated relatively marginal in our meta-analysis.

In summary, meta-analysis for the most significant compounds in EC, GEJ, and GC vs control comparison, including the healthy average concentrations (extracted from the human metabolome database) and the projected UGC concentrations in tumour tissue and plasma, resulted in several metabolites directly or inversely associated with the risk of UGC as demonstrated in Table 2. The results showed that patients with UGC compared to healthy controls especially had increased concentrations of BCAA, lysine, FAD, Acetyl-CoA, adenosine, citrulline, sphinganine, linoleic acid, putrescine, and TMAO as well as decreased concentration of asparagine, glutamine, aconitate, short-chain acylcarnitines, ATP, SM (OH) 22:1, linolenic acid, HCA, HDCA, and erythrose-4-phosphate. However, 44 out of 169 metabolites showed no significant association between patients with UGC and healthy controls through the included studies. Substantial heterogeneity (I^2^ > 50%, s2 > 0.1) was observed for some metabolites. Thus, the majority of metabolites that have been identified show inconsistent results in different studies in terms of relative abundance in the cancer versus control groups.

**Table 2:** Identified oncometabolites which resulted in consistent results among the investigated studies in patients with UGC compared to healthy controls

	SRR	Alteration	Biochemical class
TMAO	0.46	↑	Amine oxide
Isoleucine	0.35	↑	Amino acid
Leucine	0.44	↑	Amino acid
Valine	0.44	↑	Amino acid
Lysine	0.44	↑	Amino acid
Citrulline	0.36	↑	Amino acid
Asparagine	– 0.5	↓	Amino acid
Glutamine	– 0.2	↓	Amino acid
HCA	– 0.28	↓	Bile acid
HDCA	– 0.39	↓	Bile acid
Putrescine	0.65	↑	Biogenic amine
FAD	1.1	↑	Coenzym
Acetyl-CoA	1.12	↑	Coenzym
ATP	– 0.73	↓	Coenzym
Erythrose-4 phosphate	– 0.59	↓	Coenzym
Linoleic Acid	0.53	↑	Fatty acid
Short-chain Acylcarnitines			
C2	– 0.7	↓	Fatty acid
C6	– 0.43	↓	Fatty acid
C16 – OH	– 0.34	↓	Fatty acid
Linolenic acid	– 0.22	↓	Fatty acid
Adenosine	0.83	↑	Nucleoside
Aconitate	– 0.45	↓	Organic acid
Sphinganine	0.03	↑	Sphingolipid
SM (OH) 22:1	– 0.62	↓	Sphingolipid

## Discussion

Several metabolites delineated in this study were found to be significantly dysregulated in the local tumour tissue of patients with UGC as well as systemically in patients’ blood compared to healthy controls. The altered metabolites in UGC can be categorised into four main biochemical classes which affect precise oncological and patient-tailored management of UGC: carbohydrates, amino acids, lipids and nucleic acids. These classes can be again separated into several specific sub-categories considering their importance in the general cancer metabolism as well as the particular metabolism of UGC.

### Carbohydrates and metabolites of the tricarboxylic acid cycle

The glucose metabolism in esophageal- and gastric cancer cells is augmented differently from that of the normal gastric epithelium (Finley 2023). According to the Warburg effect, cancer cells support their growth primarily by generating energy via the anaerobic catabolism of glucose which implies the tumour microenvironment, in contrast to the oxidative catabolism of pyruvate in healthy cells (Warburg 1956a; Crabtree 1929). The accumulated lactic acid provides an acidic microenvironment that exacerbates the decomposition of the extracellular matrix by proteolytic activity (Bartman 2023; Lyssiotis and Cantley 2014). Several metabolites involved in cellular respiration, including lactic acid, glucose, citrate, and fumaric acid, have been frequently reported, but these results are inconsistent across studies (Cui et al. 2023; Bose et al. 2021). Moreover, opposite associations of some metabolites with UGI cancers are documented by different research groups (Matsumoto et al. 2023; Li et al. 2022; Wang et al. 2020; Wang et al. 2017; Hur et al. 2014; Ikeda et al. 2012). To our understanding, it has been reported that accumulated lactic acid moderates the activity of proteases that decompose extracellular matrix, which can produce some peptides and amino acids that are consumable for energy generation (Smyth et al. 2020; Stier et al. 2020). Acidosis microenvironment is also ascribed to the formation of cancer blood vessels, meeting the plentiful supply of nutrients and leading to tumour invasion and metastasis (Thompson et al. 2023). The contributions of these mechanistic alterations in aerobic glycolysis are crucial for understanding gastric carcinogenesis and progression (Warburg et al. 1927; Bartman et al. 2023).

Our review found ATP to be systemically decreased in patients with UGC. Because mitochondria are the primary source of ATP production, ATP is paramount in linking cancer cell metabolism and bioenergetics in the TME (Yuneva 2008). However, the hypoxic environment in cancer cells triggers the release of ATP. The in vivo measurement of extracellular ATP concentrations using e.g. cell surface–targeted *luciferase* revealed the ability of solid tumours to maintain peritumoral ATP within the range of 10^–5^ – 10^–4^ mol/l, which is much higher than the concentration of this nucleotide detected in the interstitium of healthy tissues and TME (Virgilio et al. 2018) (Marchi et al. 2019). However, because tumour cells can efficiently scavenge nucleotides, they uniquely contribute to the rapid termination of proinflammatory effects of ATP within the metabolically abnormal TME (Galon and Bruni 2019). Only the past decade has brought advances in our understanding of the complexity of mechanisms determining the duration and magnitude of purinergic signalling.

### Amino acids

Amino acids are building blocks required for cellular protein biosynthesis and cytoskeleton formation, while elevated levels of amino acids in the TME are contributing aspects in carcinogenesis (Finley 2023). Alterations in essential and non-essential proteinogenic amino acids were reported for UGI cancers (Fig. 5). The availability of amino acids is pivotal for cellular protein biosynthesis and cytoskeleton formation. At the same time, it has been pointed out that amino acids, especially those linked to TCA, are an alternative energy source of cancer cell proliferation (Smyth et al. 2020). Among them, the metabolic networks of all amino acids are complex and highly interconnected with other pathways.

Increased BCAA were consistently detected in studies on GC based on tissue and plasma samples, while inconsistent findings were obtained from studies on EC and GEJ. Several studies additionally reported altered levels of primary derivatives of amino acids in UGI cancers. Our review found significantly decreased levels of glutamine, a non-essential amino acid, in almost all studies (Faubert et al. 2014). Previous investigations delineated that metabolic regulations meet the high glutamine demand of proliferating tumour cells, which supports tumour growth by facilitating both energy production and the biosynthesis of building materials (Geldermalsen et al. 2016). However, BCAAs were found to be systemically increased in patients with UGC. Most amino acids are catabolised in the liver, except BCAAs. The liver has an active *branched-chain ketodehydrogenase* (BCKDH) to facilitate the consumption of *branched-chain ketoacids* (BCKA) for gluconeogenesis or fatty acid synthesis but is deficient in *branched-chain amino acid aminotransferase* (BCAT), the initiator of BCAA breakdown (Neinast et al. 2019). High BCAA levels may inhibit glucose metabolism, and, in turn, high glucose levels may inhibit BCAA degradation (Shao et al. 2018). Hence, abnormal amino acid metabolism has diverse and critical roles in various cancers, and the potential impact of metabolic control and regulation in the tumour microenvironment is becoming increasingly important (Sivanand and Vander Heiden 2020).

### Nucleic acids

Tumour cells are in a state of such rapid proliferation and differentiation that frequent nucleotide synthesis and metabolism are upregulated significantly. Higher uric acid or urate levels characterise accumulation of the end products of nucleotide catabolism in patients with esophageal- or gastric cancer (Wang, et al. 2021). Several studies focused on metabolites of nucleotides associated with GC and EC. However, direct evidence on the metabolic pathways of nucleotides related to UGI cancers is still unavailable (Virgilio et al. 2018). In our review, ATP has been identified to be significantly decreased in patients with UGC compared to healthy controls. The involved pathways have been delineated previously (Marchi et al. 2019).

### Lipids

The notable feature of lipid metabolism in cancer cells is an increased rate of lipogenesis and the upregulation of mitochondrial fatty acid β-oxidation. At this juncture, fatty acid oxidation provides a major energy source because complete oxidation of one 16-carbon fatty acid could generate 129 ATP molecules compared with the 38 generated from one glucose molecule (Lee et al. 2019b). Especially gastric cancer shows a similar tendency and presents typical changes regarding various metabolites involved in lipid metabolism (Lien et al. 2021). The main classes of lipids of the cell membrane, including fatty acyls, glycerophospholipids, sphingolipids, and sterols, feature their very own structure and function in the cellular membrane (Yang et al. 2022b). Respectively, the recent state of research for each biochemical class has to be delineated separately.

### Fatty acyls

Fatty acyls represent the most fundamental category of lipids. Primarily present in esterified form with glycerol, cholesterol or other lipid components, fatty acids are carboxylic acids, often with long, unbranched aliphatic chains of diverse lengths (Nguyen et al. 2014). Fatty acids are categorised as saturated (no carbon–carbon double bonds in the aliphatic chain) and unsaturated with one (monounsaturated fatty acid (MUFA)) or more double bonds (polyunsaturated fatty acid-(PUFA)) (Nomura et al. 2010). The human body can synthesise many of these fatty acids, except some essential fatty acids, including linoleic acid (omega-6 PUFA) and alpha-linolenic acid (omega-3 PUFA) (Shaw and Wolfe 1987). These two PUFAs are precursors for other omega-6 and omega-3 PUFAs that play crucial roles in regulating lipid metabolism and atherosclerosis. In our study, linoleic acid, a doubly unsaturated essential fatty acid, also known as an omega-6 fatty acid, was the fatty acid found to be associated the most with higher UGC risk, and linolenic acid, an omega-3 fatty acid, was associated with lower UGC risk. These characteristics have been described previously and are hence, consistent with the results of our investigation (Huerta-Yepez et al. 2016). However, the antitumor effect of omega-3 PUFAs and the tumour-promoting effect of omega-6 PUFAs are complex processes involving multiple factors and levels. They are interrelated, and there are still many issues to be elucidated (Lee et al. 2020).

### Glycerophospholipids

As the main subclass of phospholipids, glycerophospholipids are diacylglycerides with a phosphatidyl ester attached to the terminal carbon. The terminal ester groups are mainly ethanolamine (phosphatidylethanolamine; PE), choline (phosphatidylcholine; PC), serine (Phosphatidylserine; PS) or inositol (Phosphatidylinositol; PI) (Huybrechts, et al. 2023). In addition, several fatty acids with varying lengths and unsaturation could attach to the remaining hydroxyl groups of glycerol via either acyl-, alkyl-, or alkenyl bonds. Hydrolysis of one of the fatty acids of the phospholipids by phospholipase A2 (PLA2) generates respective lysophospholipids, adding to the diversity of the lipid pool (Behuria et al. 2022). Glycerophospholipids are the major structural component of cell membranes and are involved in various biological processes, including inflammation (Cheng et al. 2016). In the present investigation, especially phosphatidylethanolamines of different lengths have been identified to be associated with a higher risk of UGC. Our findings on alterations in PE are consistent with previous studies (Tsai et al. 2018). Furthermore, the emerging research on lipid metabolism in UGC revealed higher PC, PI and PS levels in patients with UGC compared to healthy controls (Luo et al. 2017). In Summary, these results indicate that the constellation of phosphatidylcholines, the main constituents of the cell membrane, distinctly differs between healthy cells of the upper gastrointestinal tract and tumour tissue from patients with UGC. According to the results of our meta-analysis certain phosphatidylcholines should be implicated into the diagnostic procedure of patients with UGC. At this point an investigation of the relevance of phosphatidylcholines in certain stages of the oncological treatment should be performed at pace.

### Sphingolipids

Sphingolipids are a wide range of complex lipids defined by an 18-carbon sphingoid base, usually sphingosine (SPH), found in the outer leaflet of cell membranes and in the membranous myelin sheath, which surrounds axons (Behuria et al. 2022). Condensation of SPH and free fatty acid generates the simplest sphingolipids, ceramides which function as precursors for complex sphingolipids produced by the modification of hydroxyl group with phosphocholine (in sphingomyelins) or carbohydrates (in gangliosides) (Janneh and Ogretmen 2022). Sphingolipids constitute hundreds of species originating from the combinations of varying sphingoid bases, various fatty acids that can attach to the bases and numerous carbohydrates in gangliosides. Ceramide regulates numerous cellular processes, such as proliferation, differentiation, and cell signalling (Ogretmen 2018; Zhu, et al. 2023). In UGC, there is a noticeably increased rate of lipogenesis and the upregulation of mitochondrial fatty acid β-oxidation utilising the sphingolipids to meet the demand of cell membrane synthesis, mainly for lipid raft and lipid-modified signalling molecules. The intensive fatty acid degradation via β-oxidation causes significantly larger build-ups of sphingomyelins and gangliosides in cancer tissues (Taniguchi and Okazaki 2021). Notably, in esophageal and gastric cancer tissue, there is some evidence that there are also sphingomyelins in the inner leaflet of the membrane. In addition, the overall accelerated lipid metabolism might explain the severe weight loss observed in patients with late stages of UGC (Benedetti et al. 2023). Sphinganine, a sphingoid base lipid molecule, has been identified to be increased in patients with UGC, while SM (OH) 22:1, a sphingolipid implicated in the initiation of carcinogenesis and promotion of metastasis, has been identified to be decreased in our meta-analysis. Hence, these results indicate an inconsistency in the literature (Tallima et al. 2021).

### Bile acids

Bile acids are physiological detergents that facilitate the excretion, absorption, and transport of fats and sterols in the intestine and liver, derived from catabolism of cholesterol (Fu et al. 2021). Previously, bile reflux has been shown to be an independent risk factor for precancerous gastric lesions and GC (Zhang et al. 2021). Especially, HCA a primary bile acid and HDCA, a secondary bile acid, play a crucial role in regulating energy expenditure and were both identified to be decreased in patients with UGC (Pan et al. 2022). However, our results are inconsistent with previous literature as multiple authors described primary bile acids to be reduced and secondary bile acids to be increased in patients with UGC (Rezen et al. 2022).

### Biogenic amines and related

Biogenic amines are mainly produced by the breakdown or transformation of amino acids, typically containing one or more amino groups. As such, they comprise a wide range of very heterogeneous metabolites involved in various cellular processes (Fernandez-Reina et al. 2018). A much-discussed metabolite which has been demonstrated to be a prerequisite for cell proliferation to occur is Trimethylamine N-oxide (TMAO). There is evidence suggesting that higher levels of TMAO and its precursors in blood can indicate either a higher risk of malignancy or its presence (Stonans, et al. 2023). TMAO is anticipated to have significance as a biomarker of—or even an independent risk factor for UGC (Oellgaard et al. 2017). More studies confirmed the association of microbiota with TMAO production, resulting in higher risk of colorectal, breast, and gastric cancer (Khodabakhshi et al. 2023). Another metabolite in this heterogenous field is the polyamine putrescine, a polycationic alkylamine commonly found in all living cells and essential for cellular growth and survival. Physiologically, it is sequentially synthesised from the catabolism of the amino acids L-arginine and L-methionine (Janne et al. 2004). However, the putrescine concentrations were significantly increased in patients with UGC and their TME compared to the adjacent normal gastric mucosa from the same individuals (McNamara et al. 2021). Respectively, with the inhibition of the growth of human gastric tumour; putrescine levels were significantly decreased in the tumour tissue (Xie et al. 2023). Consistent with previous findings, we found both TMAO and putrescine to be significantly increased in patients with UGC.

The majority of metabolites that have been identified show inconsistent results in different studies in terms of relative abundance in the cancer and its microenvironment versus control groups. Accordingly, it infers that cancer cells gain growth superiority over their regular counterparts by switching metabolic energy patterns to anaerobic glycolysis and possibly fumarate respiration instead of securing more ATP. These studies, including ours, collectively support that understanding the metabolic risk of many malignancies, including esophageal cancer, cancer of the gastroesophageal junction, and gastric cancer, could improve the development of precise oncological and patient-tailored management of UGC (Han and Lee 2024). Overall, these data justify the evaluation of existing models of amino acid, lipid, carbohydrates and nucleic acid metabolism and their role in tumour growth, metastasis, and immunosurveillance. The measurement of soluble and exosomal activities in the blood of cancer patients might represent a compelling addition to the ‘liquid biopsy’ arsenal (Jaras et al. 2023). Although the described literature reports elevated levels of several oncometabolites such as acetylcarnitine (C2), hexadecanoylcarnitine (C16), octadecenoylcarnitine (C18:1), hydroxytetradecadienylcarnitine (C14:2–OH), and octadecanoylcarnitine (C18) in patients with UGC, our comprehensive meta-analysis encompassing a larger dataset did not corroborate these findings. This discrepancy suggests a need for further investigation to reconcile these conflicting results and to understand the underlying factors contributing to these differences (Corona, et al. 2018; Lario et al. 2017).

### Strengths and limitations

To the best of our knowledge, our study provides the most comprehensive meta-evidence on the associations of single metabolites with UGC. This systematic review’s particular strength is the large number of included studies conducted in different populations and the adoption of metabolomics profiling using various biospecimens and analytic techniques. We have performed meta-analysis for a large number of metabolites to facilitate the development of precise oncological and patient-tailored management of UGC. Narrowing the review’s focus to studies with open-access data allowed us to limit the influence of reverse causality and selection bias, and most of the included studies were evaluated to be of high quality.

There are several limitations to be considered. First, our search covered only four databases. However, relevant studies are usually indexed in those databases. Second, the majority of metabolites for which we performed meta-analysis showed considerable between-study heterogeneity and resulted in inconsistent results in our meta-analysis. Possible reasons why certain oncometabolites show inconsistent results may lie both in the pre-analysis and in the analysis of the respective study. The metabolites ATP, UTP and NADH, for example, reflect metabolites that can be measured in different concentrations even when there are small differences in the pre-analytical procedures, such as different biopsy sampling or lyophilization due to the energetic instability of the cells after biopsy sampling (Jang et al. 2018; Tomita et al. 2012). Third, the observational nature of the studies included in the meta-analysis impedes inferences about potential causal mechanisms underlying the observed UGC associations or the histology of particular UGC such as the differentiation of GC in adenocarcinoma and signet-cell carcinoma. Forth, in this meta-analysis, only metabolites were considered that exhibited a relevant difference between tumor patients and healthy controls both in local tumor tissue and systemically in blood plasma and can therefore actually be classified as oncometabolites. However, the investigation was not subdivided into the separate entities of UGCs. In this respect, a further limitation of the study is that metabolites that are exclusively elevated locally in tumor tissue or exclusively elevated systemically in blood plasma were not investigated in detail. Nevertheless, a subgroup analysis of metabolites that are elevated locally in tumor tissue or exclusively systemically should be performed in future studies. We only considered evidence from metabolomics studies, which implies that we disregarded potentially relevant data from targeted assays for selected metabolites by design. In this respect, it is crucial that methods currently experiencing significant interest in oncometabolomics research should also be considered in a subsequent investigation. These methods particularly include, matrix-assisted laser desorption ionization (MALDI) – mass spectrometry imaging (MSI) and the field of spatial metabolomics.

Also, we did not study the relationship between the identified metabolites and oncological outcome parameters such as the response to chemotherapy within each study and the relationship with the TNM – or the UICC classification. However, the necessity of an investigation of the relationship between the metabolome and oncological outcome parameters has been highlighted by the results. Finally, misclassification of the reports during study selection and publication bias are essential sources of bias for evidence summaries, which we mitigated through an independent review of the included studies by three authors and detailed analyses of publication bias.

## Conclusion and perspectives

The present systematic review and meta-analysis provides an updated overview of the associations between many metabolites and UGC. We performed a meta-analysis for 169 tissue and plasma metabolites associated with the risk of UGC, detecting 155 significant risk associations. The UGC–associated compounds reflect dysregulation of various processes, such as proteolysis, gluconeogenesis, mitochondrial function, and fatty acid oxidation. Many discrepancies were found between the studies, for example, in metabolite behaviour. A small number of studies in each group correctly evaluated the results. The necessity of an investigation of the relationship between the metabolome and oncological outcome parameters has been highlighted by the results of this systematic review and meta-analysis.

Although the patients included in these studies are from across the globe, a targeted multicentric study is necessary to develop a precise oncological and patient-tailored management of UGC. Another point that should be considered is the analysis and research of the same cohort with techniques to account for metabolomics compounds. Finally, the reproduction of some of these studies would enable the use of these metabolites as biomarkers and is highly desirable. We encourage the metabolomics communities to fully disclose their ethics approvals and samples’ storage conditions, report compound names with at least one identifier and include p-values and fold changes for the relevant compounds. With additional investigations published by the scientific community, some of the relevant metabolites found might be reaffirmed as applicable, and others may become irrelevant for UGC pathophysiology. According to the results of our meta-analysis especially BCAA and TMAO as well as certain phosphatidylcholines should be implicated into the diagnostic procedure of patients with UGC. At this point an investigation of the relevance of phosphatidylcholines in certain stages of the oncological treatment should be performed at pace.

## Key messages

### What is already known on this topic

The prognosis of patients with upper gastrointestinal cancers, i.e. esophageal cancer, cancer of the gastroesophageal junction, and gastric cancer (UGC), is poor in advanced stages. The concept of metabolic plasticity in tumours and their tumour microenvironment (TME) has recently emerged as we started to realize that tumours alter their metabolism to meet the high demands of energy and biomass to fuel their rapid growth and metastasis. In this study, we aimed to characterize differences in concentrations of plasma and tissue metabolites across patients with UGC and healthy controls. Using a statistical approach for vague clustering, we classify groups of metabolites into biochemical and functional categories.

### What this study adds

This research summed up the metabolites previously identified to be either increased or decreased in patients with UGC in a systematic review and meta-analysis to create a metanarrative of the current association between metabolites and UGC. In conclusion, summarizing the metabolic profile of patients with upper GI tumours using LC-MS/MS and 1H NMR spectroscopy resulted in the identification of an upper GI cancer-specific signatures driven by increased concentrations of BCAA, lysine, FAD, Acetyl-CoA, adenosine, citrulline, sphinganine, linoleic acid, putrescine, and TMAO as well as decreased concentration of asparagine, glutamine, aconitate, short-chain acylcarnitines, ATP, SM (OH) 22:1, linolenic acid, HCA, HDCA, and erythrose-4-phosphate in plasma, tumour tissue and tissue from the TME compared to healthy controls. The necessity of an investigation of the relationship between the metabolome and oncological outcome parameters has been highlighted by the results of this systematic review and meta-analysis.

### How this study might affect research, practice or policy

These data demonstrate the promising potential for plasma and tissue metabolome analyses to dissect the molecular mechanisms of metabolic plasticity of tumours and develop a precise oncological and patient-tailored management of UGC as well as a first approach to metabolism-based cancer therapy. However, further analyses with larger patient cohorts are required to validate this observation. According to the results of our meta-analysis especially BCAA and TMAO as well as certain phosphatidylcholines should be implicated into the diagnostic procedure of patients with UGC.

## Supplementary Information

Below is the link to the electronic supplementary material.

Supplementary file1 (XLSX 42 KB)

## Data Availability

All the data are provided in the supplements.

## Abbreviations

EC: Esophageal cancer
GEJ: Cancer of the gastroesophageal junction
GC: Gastric cancer
UGC: Upper gastrointestinal cancers
PRISMA: Preferred Reporting Items for Systematic Reviews and Meta-Analyses
PROSPERO: International Prospective Register of Systematic Reviews
NOS: Newcastle–Ottawa Scale
QUADOMICS: An adaptation of the QUADAS-2 (Quality Assessment of Diagnostic Accuracy)
BCAA: Branched-chain Amino Acids
BCKDH: Branched-chain alpha-Keto Acid Dehydrogenase Complex
BCAT: Branched-chain Amino Acid Aminotransferase
NMR: Nuclear magnetic resonance spectroscopy
MS: Mass spectroscopy
LC: Liquid Chromatography
GC: Gas Chromatography
CE: Capillary Electrophoresis
LC–MS/MS: Liquid Chromatography-Tandem Mass Spectroscopy
HMDB: The Human Metabolome Database
UPLC-MS/MS: Ultra-Performance-Liquid Chromatography-Tandem Mass Spectroscopy
HPLC-GC/MS–MS: High-Performance-Liquid Chromatography-Tandem Mass Spectroscopy
GI: Gastrointestinal
1H NMR: Proton Nuclear Magnetic Resonance or Hydrogen-1 NMR
UPLC-TQ-MS: Ultra-Performance Liquid Chromatography-Triple Quadruple Mass Spectrometry
UHPLC-MS: Ultra-Performance Liquid Chromatography- Mass Spectrometry
UHPLC-Q-TOF/MS: Ultra-High Performance Liquid Chromatography-Quadrupole Time-of-Flight Mass Spectrometry
CE-TOF/MS: Capillary Electrophoresis Time-of-Flight Mass Spectrometry
GC/TOF–MS: Gas Chromatography–Time-of-Flight Mass Spectrometry
HPLC/ESI/Q-TOF/MS: High-Performance Liquid Chromatography coupled to Electrospray Ionization Quadrupole Time-of-Flight Mass Spectrometry
UPLC–TOF/MS: Ultra-Performance Liquid Chromatography- Time-of-Flight Mass Spectrometry
MRB–CE–MS: Moving Reaction Boundary Capillary Electrophoresis-Mass Spectrometry
MALDI MS: Matrix-Assisted Laser Desorption/Ionization- Mass Spectrometry
MALDI MSI: Matrix-Assisted Laser Desorption/Ionization- Mass Spectrometry Imaging
SIFT-MS: Selected Ion Flow Tube Mass Spectrometry
SPME-GC/MS: Solid-Phase Microextraction-Gas Chromatography-Mass Spectrometry
HR-MAS NMR: High-Resolution Magic Angle Spinning-Nuclear Magnetic Resonance
InChIKey: International Chemical Identifier
CTS service: Chemical Translation Service
FAD: Flavin Adenine Dinucleotide
SM: Sphingolipids
HCA: Hyocholic acid
HDCA: Hyodeoxycholic acid
TCA: Tricarboxylic acid cycle
MUFA: Monounsaturated Fatty acid
PUFA: Polyunsaturated Fatty acid
MG: Monoacylglycerol
DG: Diacylglycerol
TG: Triacylglycerol
PLA2: Phospholipase A2
Me: Means in experimental group
Se: Standard deviation in experimental group
Ne: Headcount in experimental group
Mc: Means in control group
Sc: Standard deviation in control group
Nc: Headcount in control group
